# Compartment-Specific Amino Acid Dysregulation in Depression: Integrating Non-Targeted Metabolomics with Brain Barrier Biology

**DOI:** 10.3390/ijms27219847

**Published:** 2026-11-03

**Authors:** Ivana Perić, Andrijana Stanisavljević Ilić, Neda Đorđević, Nevena Todorović Vukotić

**Affiliations:** Department of Molecular Biology and Endocrinology, Vinča Institute of Nuclear Sciences—National Institute of the Republic of Serbia, University of Belgrade, Mike Petrovića Alasa 12-14, 11351 Belgrade, Serbia; andristanisavljevic@vinca.rs (A.S.I.); neda@vin.bg.ac.rs (N.Đ.); nevenat@vin.bg.ac.rs (N.T.V.)

**Keywords:** depression, metabolomics, amino acid, MetaboListem, blood–brain barrier, amino acid transporter

## Abstract

The introduction of metabolomics into depression research, together with advances in bioinformatics, has substantially expanded our understanding of molecular alterations associated with this disorder. However, the rapid growth of metabolomic studies, combined with variability in analytical workflows, biological matrices, and experimental models, presents major challenges for data interpretation and cross-study integration. In this article, we systematically summarized reported metabolite alterations in human and animal studies using a combined natural language processing (NLP)-based text-mining and vote-counting approach. To our knowledge, this is one of the first studies to apply NLP-based abstract mining to depression-related metabolomics literature across multiple biological compartments. Text-mining analysis of publications retrieved from PubMed and Scopus identified proteinogenic amino acids as the most consistently reported class of altered metabolites across peripheral and central matrices, followed by lipids and other metabolic categories. Vote-counting analysis of non-targeted metabolomics studies revealed relatively consistent decreases in circulating large neutral and branched-chain amino acids, with Leu, Met, Trp, Tyr, and Val representing the most consistently overlapping alterations across human and animal studies. Finally, these findings were discussed in the context of brain barrier transport systems, considering altered metabolite exchange across the blood–brain interfaces as one of several possible explanations for the compartment-specific amino acid alterations observed in depression.

## 1. Introduction

Depression is a prevalent mental disorder characterized by persistent low mood and loss of interest or pleasure in daily activities, affecting approximately 5% of adults globally, according to the World Health Organization. It represents one of the leading causes of disability worldwide and imposes a substantial burden on individuals, healthcare systems, and work productivity [1]. Current evidence suggests that the etiology of depression involves a complex interplay between genetic susceptibility and environmental factors. Despite the availability of multiple antidepressant treatment strategies, primarily based on pharmacotherapy, many patients fail to achieve clinically meaningful improvement or full remission. Moreover, meta-regression analyses indicate that treatment outcomes in usual care have not substantially improved over time, with approximately 80% of patients failing to achieve a clinical response, highlighting the need for a better understanding of the underlying biological mechanisms [2].

Beyond alterations in neuronal signaling and immune regulation, increasing evidence suggests that disturbances in mechanisms maintaining brain homeostasis may contribute to depression pathophysiology. Biological barriers, including the blood–brain barrier (BBB) and blood–cerebrospinal fluid (CSF) barrier, represent critical interfaces regulating molecular exchange between peripheral circulation and central compartments, including CSF and brain tissue. Alterations in barrier integrity and transport capacity may influence the distribution and availability of bioactive molecules, thereby affecting neurotransmission, energy metabolism, and neuroplasticity. Although barrier alterations have been investigated in several neurological and psychiatric disorders, their contribution to metabolic disturbances observed in depression remains insufficiently characterized. Importantly, metabolite profiles obtained from different biological compartments may reflect not only systemic metabolic alterations but also compartment-specific regulation, molecular transport processes, and changes in central metabolic homeostasis.

Recent advances in state-of-the-art omics technologies, together with developments in bioinformatics and machine learning (ML), offer promising avenues for elucidating the biochemical underpinnings of depression. Nevertheless, a universally accepted or clinically robust molecular signature for diagnosis, prognosis, or treatment monitoring remains elusive. Furthermore, the increasing number of metabolomics studies has created a need for systematic approaches capable of integrating heterogeneous datasets and identifying reproducible metabolic patterns across biological compartments.

## 2. Metabolomic Phenotyping

Metabolomics is an experimental approach that enables the identification and quantification of small molecules, typically with molecular masses below 1500 Da, within biological samples [3]. Over the past two decades, metabolomics has been increasingly applied to the study of brain disorders, with early investigations laying the groundwork for its integration into neuropsychiatric research [4,5,6]. When combined with data science and bioinformatics, metabolomics provides a powerful framework for elucidating the biochemical mechanisms underlying complex brain processes and disease states. A central objective of this integrative approach is the identification of specific metabolites that may serve as informative biomarkers, thereby supporting the diagnosis of neuropsychiatric disorders, the evaluation of disease severity, and the assessment of therapeutic efficacy [7].

Compared with other omics approaches, metabolomics has a unique capacity to capture real-time biochemical dynamics, reflecting cellular responses and phenotypic interactions while integrating both genetic and environmental influences. Because metabolites represent the final products of biochemical processes, they are closely linked to observable phenotypic outcomes, including clinical symptoms. This feature makes metabolomics particularly well suited for investigating treatment responses and their behavioral correlates. Supporting this notion, a recent study comparing metabolomic and polygenic risk scores demonstrated that metabolomic scores were more strongly associated with disease onset, including depression, and provided greater predictive power for identifying high-risk individuals [8].

**Figure 1:**
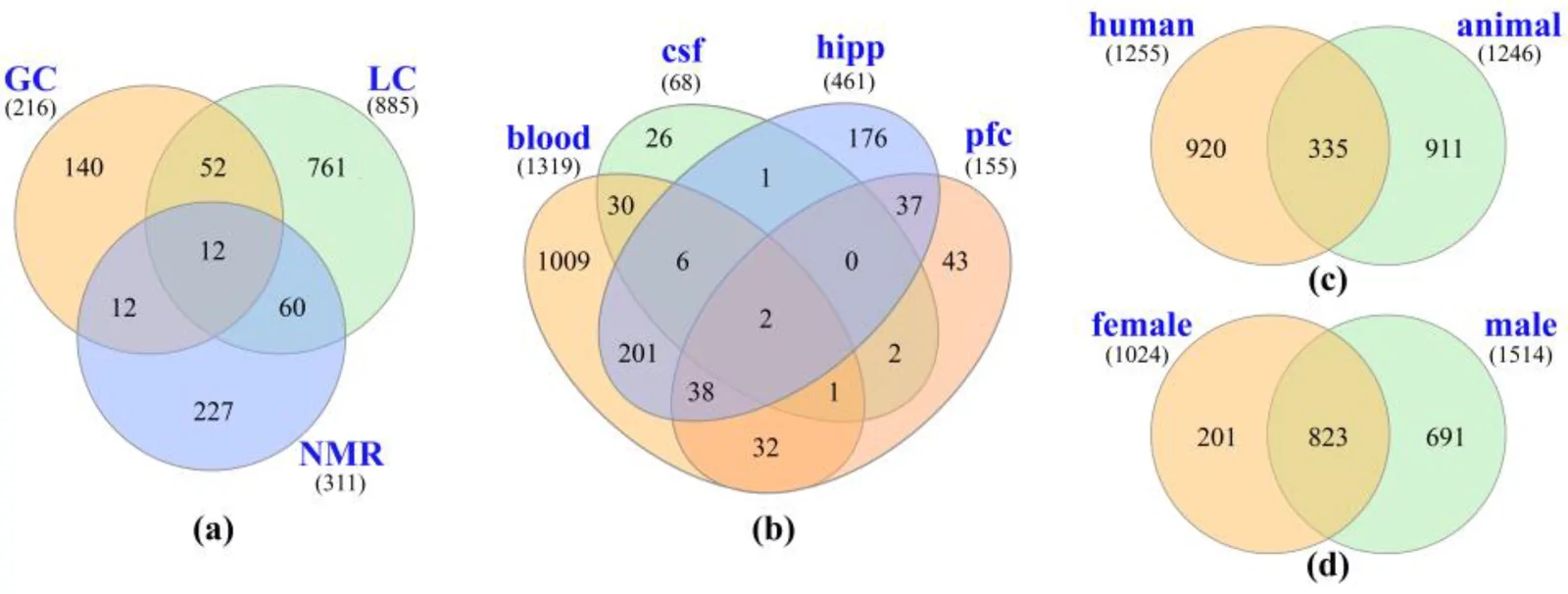
Summary of methodological diversity in metabolomic studies of depression. Venn diagrams were generated using InteractiVenn v1 [12] to illustrate the distribution of studies according to (**a**) analytical techniques, (**b**) biological sample types, (**c**) study models (human versus animal), and (**d**) sex distribution across studies. Data were retrieved from the PubMed and Scopus databases on 22 October 2026.

Figure 1 provides a general overview of how metabolomics has been applied in depression research. It highlights the broad range of study designs, encompassing both human studies and animal models, as well as the diversity of analytical platforms employed, including gas chromatography (GC), liquid chromatography (LC), and nuclear magnetic resonance (NMR). In addition, the figure illustrates the most frequently investigated biological matrices in depression-related metabolomic studies, such as blood (serum and plasma), CSF, and brain regions including the hippocampus (HIPP) and prefrontal cortex (PFC). Collectively, these studies underscore the considerable methodological heterogeneity within the field. While this diversity enables a comprehensive exploration of the depression metabolome, it also poses substantial challenges for cross-study comparability and the identification of consistent biomarkers across tissues and species. The strengths and limitations of these analytical approaches have been discussed previously [9,10,11].

The growing availability of metabolomics databases and advanced analytical platforms has substantially expanded the capacity to process large-scale datasets. These resources have facilitated the widespread adoption of non-targeted metabolomics approaches, which are now essential for constructing disease- or treatment-specific metabolic profiles and for identifying perturbed biological pathways. With access to hundreds of thousands of metabolite spectra, researchers are increasingly able to uncover novel insights into the biochemical basis of depression. Despite these advancements, metabolomic research remains challenged by the inherent sensitivity of metabolites to a wide range of internal and external variables. This sensitivity underscores the critical importance of stringent experimental control to ensure data reproducibility.

In animal studies, even subtle methodological differences, such as combining samples from different experimental batches, treating animals at different times, or having different personnel conduct the experiments, can introduce substantial variability that may obscure true biological effects. Empirical evidence therefore emphasizes the importance of using animals from the same batch and maintaining highly standardized experimental conditions to generate consistent and reliable metabolomic data. Although human studies are inevitably influenced by uncontrollable environmental and biological variability, animal models remain invaluable in metabolomics research because experimental parameters can be tightly controlled and defined conditions replicated.

Previous efforts have been made to synthesize metabolomic findings in depression, including integrated meta-analyses of peripheral blood metabolites and their biological functions by Pu et al. and Panina et al. [13,14]. As the metabolomics literature continues to expand, complementary approaches are also valuable for systematically screening the available evidence [15]. Text mining enables large-scale automated data extraction and provides a rapid means of mapping reported metabolic alterations across a large body of literature, although it does not replace quantitative meta-analysis or provide equivalent statistical evidence. In this context, natural language processing (NLP) technologies are gaining attention as emerging tools in bioinformatics. NLP-based approaches have been developed to support the extraction and interpretation of biomedical knowledge from scientific literature [16,17,18,19,20,21]. Although still under active development for domain-specific applications such as metabolomics, NLP-based approaches show early promise in identifying molecular patterns and disease mechanisms through automated literature analysis [16,22].

Notably, specialized algorithms such as MetaboListem, designed for metabolite name recognition, have demonstrated high performance, supporting their potential utility in large-scale mining of metabolomics literature [23]. In the present review, NLP-based text mining of abstracts using the online tool MetaboListem was applied as an exploratory, hypothesis-generating approach to identify frequently reported metabolites and metabolic classes in depression-related metabolomics studies. The results guided focused manual curation and directional vote-counting of reported amino acid alterations across multiple biological compartments, distinguishing findings from human and animal studies.

## 3. Methodology

We conducted a systematic review in accordance with the PRISMA 2020 guidelines [24], incorporating NLP-based text-mining and a vote-counting strategy, by searching the PubMed and Scopus databases for relevant literature.

### 3.1. Inclusion and Exclusion Criteria for NLP-Based Text Mining Analysis

**Figure 2:**
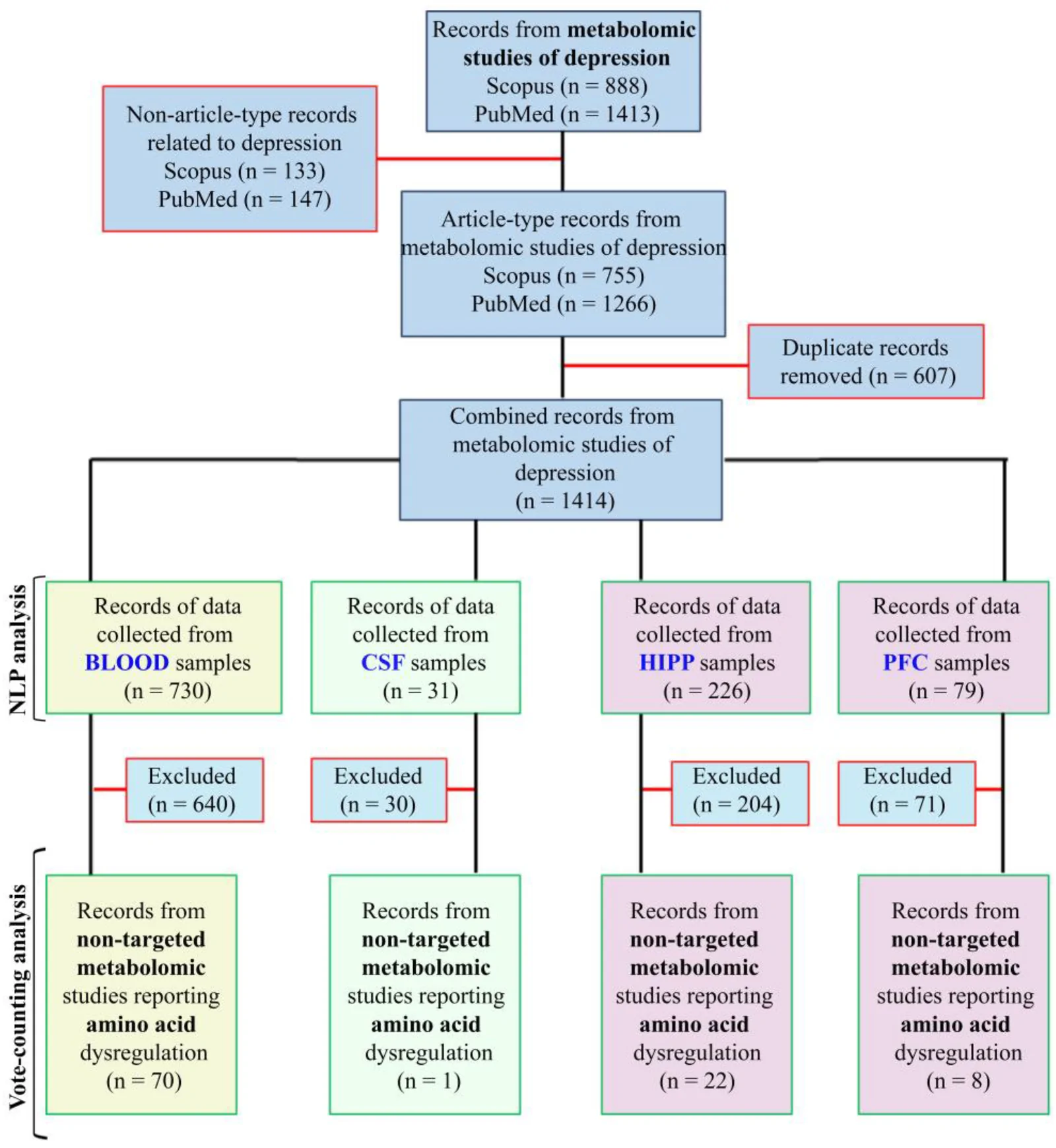
Flowchart illustrating the two-stage study selection and analysis process. Stage 1: MetaboListem-assisted NLP-based literature mapping; Stage 2: manual eligibility screening and vote-counting analysis.

The inclusion criteria were peer-reviewed articles with assigned DOI numbers, without restrictions on publication year or language, reporting clinical and preclinical studies employing metabolomic techniques. The search strategy (Table S1), based on the search sequence (depression OR depressive) AND (metabolomic OR metabolome) AND (serum OR plasma OR blood OR “cerebrospinal fluid” OR hippocampus OR hippocampal OR “prefrontal cortex”), was applied to article titles, abstracts, and keywords. Review articles were excluded from the NLP analysis. Figure 2 presents the flow diagram of study selection and inclusion in the NLP-assisted frequency-based analysis. All articles published up to 25 December 2025 were considered in the review. Study selection and data extraction were performed manually by the authors, who reviewed the identified publications and extracted information relevant to the subsequent analyses. A formal risk-of-bias assessment was not performed. Because the review focused on non-targeted metabolomic studies and reporting practices varied across publications, the reasons for unreported metabolite findings could not be reliably determined.

### 3.2. NLP-Based Text-Mining Analysis

Text mining was performed using MetaboListem, an open-source metabolite name recognition tool based on named entity recognition, implemented in Python 3.7 and available at https://github.com/omicsNLP/MetaboliteNER (accessed on 16 April 2025). MetaboListem allows for the simultaneous search of nearly 160,000 metabolite name variants across large datasets, with an F1-score of 0.8900, precision of 0.8923, and recall of 0.8877 [23].

To our knowledge, this review represents the first application of MetaboListem in a literature-based analysis of depression-related metabolomics research. The text-mining analysis produced a list of metabolites, with each metabolite counted only once per abstract and its frequency reflecting the number of abstracts in which it was identified. A notable limitation of the NLP analysis was the inconsistency in how metabolites were reported across studies, often resulting in redundant entries for the same compound. To address this, we manually reviewed and standardized the top 60 most frequently identified metabolite names. Metabolite names obtained through NLP-based literature mining were manually post-processed to consolidate synonyms referring to the same metabolite (e.g., glutamate and glutamic acid). For each group of synonymous names, the most frequently occurring name was retained as the final name, and the frequency counts of synonymous names were summed. These curated results were then used to perform enrichment and pathway analysis and to guide a targeted manual review of the literature.

The text mining complied with the Terms of Service and TDM policies of the respective databases. Only abstracts and metadata were used, and no full-text articles protected by copyright were downloaded or redistributed. No third-party figures or tables were reused in the present study. The NLP tool used is disclosed (name, version, and whether custom-built or commercial).

### 3.3. Enrichment and Pathway Analysis

To explore the biological relevance of the most frequently reported metabolite alterations across studies, metabolite set enrichment and pathway analyses were performed using MetaboAnalyst version 6.0 (https://www.metaboanalyst.ca/ accessed on 16 April 2025). The list of the 60 most frequently reported metabolite alterations was uploaded for analysis.

Metabolite set enrichment analysis was performed using over-representation analysis with the hypergeometric test and the Small Molecule Pathway Database (SMPDB). Compounds were entered by name, with the input type set to significant features and the feature type specified as metabolites. Metabolite sets containing at least two entries were considered. All compounds in the selected library were used as the reference metabolome.

Pathway analysis was conducted using compound names as input, with the input type set to significant metabolites and the pathway library set to Homo sapiens (KEGG). The hypergeometric test was used as the enrichment method, relative betweenness centrality as the topology measure, and the scatter plot as the visualization method. All compounds in the selected pathway library were used as the reference metabolome.

Interpretation primarily focused on metabolite sets and pathways with false discovery rate (FDR)-adjusted *p*-values ≤ 0.05. Pathways with an impact score of 1.0 were also considered as exploratory findings, even when they did not meet the statistical significance threshold, and were interpreted cautiously in view of their potential mechanistic relevance.

### 3.4. Vote-Counting Strategy

Building upon the results of NLP-based text mining, a vote-counting strategy was applied to obtain a more detailed and structured overview of metabolic alterations associated with depression. Given that amino acids emerged as the most frequently reported and dominant molecular class in the text-mining analysis, vote counting was specifically conducted for proteinogenic amino acid alterations. In addition to the vote-counting analysis of proteinogenic amino acids, GABA was included in the review as a non-proteinogenic amino acid frequently reported as altered in depression-related metabolomic studies. Its inclusion also reflected its metabolic relationship with glutamate, mediated by glutamate decarboxylase, and its role as the major inhibitory neurotransmitter in the central nervous system. This focused approach was chosen to enable a more robust evaluation of directional consistency while limiting methodological heterogeneity. Vote counting was restricted exclusively to findings derived from non-targeted metabolomics studies, with the aim of constructing an integrated framework of depression-related amino acid alterations and assessing the consistency and variability of non-targeted results across the literature.

The records retrieved from the PubMed and Scopus databases using the search strategy described above were first subjected to NLP-based analysis. The same records were then further screened according to additional exclusion criteria to identify studies eligible for vote counting. Additional exclusion criteria included: targeted metabolomic studies; non-targeted metabolomic workflows accompanied by targeted data analysis (e.g., metabolite annotation restricted to in-house libraries); meta-analyses; studies focusing on bipolar disorder or other disease comorbidities; case reports; and publications without accessible full text. The authors manually reviewed the identified publications and extracted reported amino acid alterations, their direction of change, the biological matrix examined, the study model (human or animal), the animal model of depression, and the instrumental approach. Findings for which the direction of change could not be determined were excluded from the vote-counting analysis. A flow diagram depicting the study selection and inclusion process for the vote-counting analysis is presented in Figure 2. Following the application of the exclusion criteria, the final numbers of studies included in the vote-counting analysis were 70, 1, 22, and 8 for the blood, CSF, HIPP, and PFC matrices, respectively.

Non-targeted metabolomic studies apply different criteria for selecting and reporting results, including statistical significance, variable importance in projection (VIP) scores, fold-change thresholds, and metabolite identification or annotation criteria. Consequently, the absence of an amino acid from a study’s reported findings could not be interpreted as evidence of a non-significant result. Because non-significant findings and unreported metabolites could not be systematically identified across studies, they were not included in the vote-counting analysis. For each amino acid, the numbers of significant reported increases and decreases were summarized separately for each biological matrix and study type (human or animal). Directional consistency was calculated as the proportion of significant reported findings in a given direction relative to the total number of significant reported increases and decreases for that amino acid within the specified matrix and study type. Thus, the denominator comprised all significant reported increases and decreases for the respective amino acid, biological matrix, and study type.

Amino acids for which at least 70% of significant reported findings showed the same direction of change were classified as consistently increased or decreased. Directional-consistency classifications were made only when findings from at least two independent studies were available for the respective amino acid, biological matrix, and study type. To assess the robustness of these classifications, a sensitivity analysis was performed using alternative thresholds of 60%, 70%, and 80%. The original 70% threshold was retained for the primary analysis and corresponding figures, and the same minimum-evidence requirement was applied across all thresholds.

## 4. Text-Mining Results for Metabolite Dysregulations Reported in Depression Research

**Figure 3:**
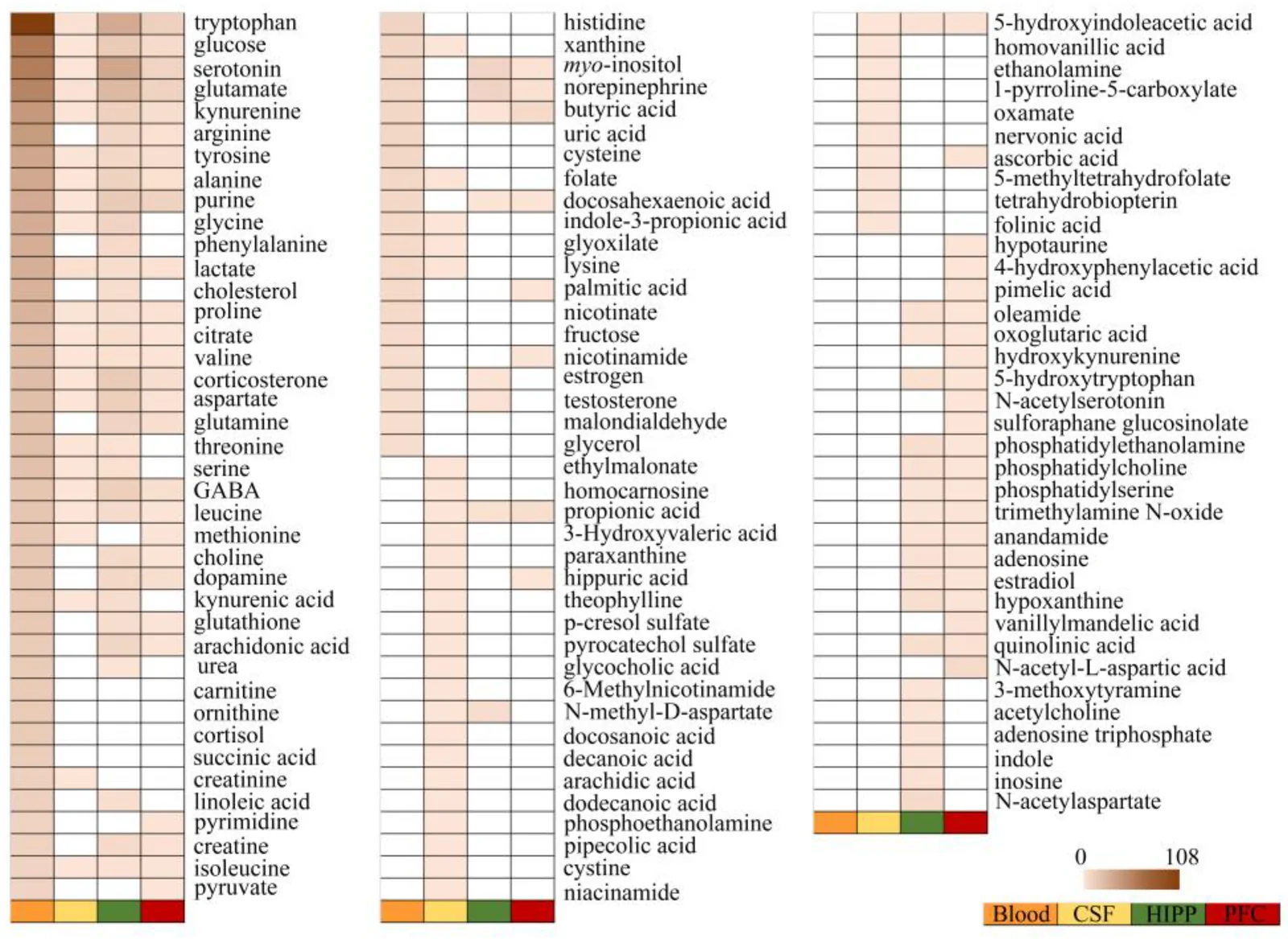
MetaboListem-derived analysis of metabolomic alterations associated with depression across blood, CSF, HIPP, and PFC. The heatmap illustrates the top 60 most frequently reported metabolites in each biological matrix, as identified through text mining of depression-related studies.

The initial text-mining analysis identified 1493, 94, 530, and 220 distinct metabolite entities reported as altered in blood, CSF, HIPP, and PFC, respectively, in depressive conditions. Following manual post-processing, the 60 most frequently reported metabolites across multiple tissues are summarized in Figure 3.

### Enrichment and Pathway Analysis

To further explore the biological interpretation of the most frequently reported metabolomic alterations associated with depression, metabolite set enrichment and pathway analyses were performed on the most frequently reported metabolites identified across the reviewed studies. The goal was to identify common biochemical pathways that may contribute to the pathophysiology of depression. The results, including the top-enriched metabolite classes and the most significantly affected pathways across blood, CSF, and brain regions, are presented in Figures S1–S4. These analyses aimed to highlight shared biochemical mechanisms and pathways that may underlie depression-related metabolic alterations across different biological matrices.

## 5. Amino Acid Dysregulations in Non-Targeted Metabolomic Studies of Depression

Text-mining and bioinformatic analyses indicate that metabolite alterations in depression tend to cluster by metabolite class, highlighting the involvement of shared biological pathways in the depressive phenotype. Amino acids emerged as the most consistently dysregulated metabolite class across all four examined biological matrices (Figure 3). Complementary pathway-based enrichment analyses further support this observation (Figures S1–S4).

The vote-counting results provide a summary of the directional consistency among significant reported findings of proteinogenic amino acid alterations in non-targeted metabolomic studies across different biological matrices, offering insights into the consistency and variability of these changes in both human and animal research.

### 5.1. Amino Acid Dysregulation in Blood-Derived Samples

Blood reflects complex interactions among multiple organ systems and serves as a reservoir of biomarkers indicative of metabolic state, organ function, and pathology [10,11]. Serum metabolomic profiles have been reported as predictive of major depressive disorder and other conditions [8,13,25]. Study-level findings on amino acid alterations reported in blood-derived samples are summarized in Table S2, while the results of the vote-counting analysis are presented in Table 1.

**Figure 4:**
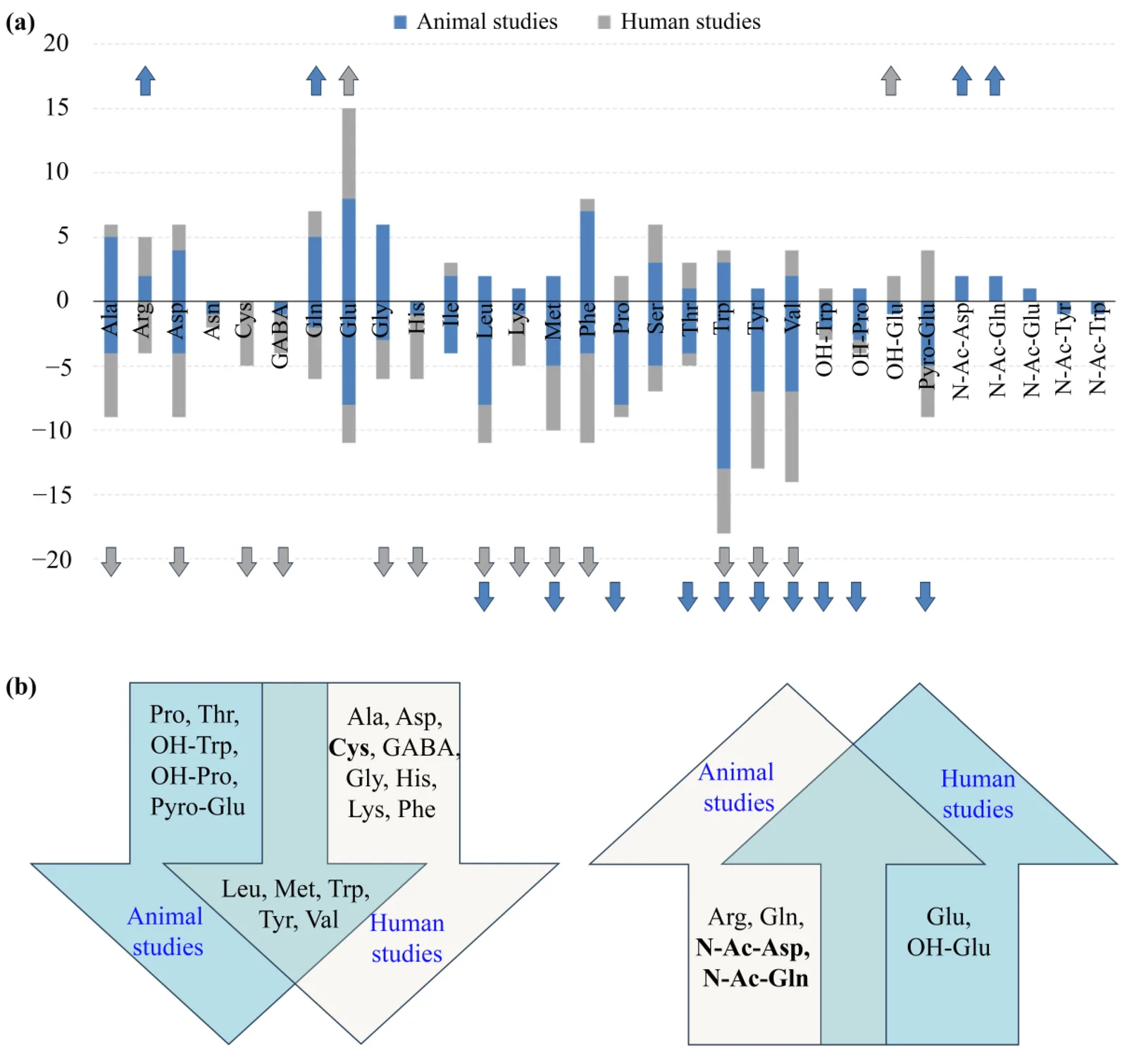
(**a**) Stacked bar representation of directional consistency among significant reported findings of amino acid alterations in blood according to study type. Bars indicate the number of independent non-targeted metabolomic studies reporting significantly increased (positive values) or significantly decreased (negative values) levels for each metabolite in human and animal studies of depression. Human and animal studies are displayed as separate segments of each stacked bar. Directional consistency (≥70% of studies reporting the same direction across ≥2 independent studies) was assessed independently for human and animal studies, with blue arrows representing animal studies and gray arrows representing human studies; (**b**) Schematic overview of directional consistency among significant reported findings depicting increases and decreases in blood amino acid levels. For each direction of change, two arrows are shown for human and animal studies; overlapping sections indicate amino acids consistently altered in both clinical and preclinical studies, whereas non-overlapping sections represent metabolites showing consistent changes only in human or animal studies. Metabolites shown in bold denote unambiguous alterations (100% agreement among all studies within the respective human or animal dataset).

The amino acids shown in Figure 4 were classified based on the primary 70% directional-consistency threshold, with the robustness of these classifications assessed through sensitivity analysis using alternative thresholds of 60% and 80% (see Table S3). Despite some variability across studies, consistent directional trends are observed for several proteinogenic amino acids, as well as for γ-aminobutyric acid (GABA). In addition to text-mining summaries, vote-counting analyses indicate that each of the 20 proteinogenic amino acids has been reported as altered, with specific amino acids showing reproducible increases or decreases across independent studies reporting statistically significant changes. Overall, while heterogeneity exists, the observed consistency for a subset of amino acids suggests a robust pattern of peripheral metabolic alterations associated with depression.

As summarized in Figure 4a, the most consistently reported decreases in blood amino acid levels included Leu, Met, Trp, Tyr, and Val across all studies. Several amino acids were consistently reported as decreased only in human studies, without reproducible alterations in animal models; these included Ala, Asp, Cys, GABA, Gly, His, Lys, and Phe. Conversely, decreases consistently observed only in animal studies included Pro, Thr, OH-Trp, OH-Pro, and pyro-Glu. Overall, given the substantial variability across studies, only a limited subset of amino acids exhibited unambiguous changes (i.e., showed a directional consistency among significant reported findings), including Asn, Cys, GABA, His, and N-acetylated amino acid derivatives.

With respect to increased blood amino acid levels, significant reported findings were limited and showed no overlap between human and animal studies (Figure 4b). Specifically, human studies consistently reported increases only in Glu and OH-Glu, whereas animal studies showed consistent increases in Arg, Gln, N-acetyl-Asp, and N-acetyl-Gln. These results indicate that depression-associated elevations in circulating amino acids lack cross-species consistency, in contrast to the decreases, which show partial overlap between human and animal studies.

The sensitivity analysis (Table S3) revealed that most human findings remained consistent under the more stringent 80% threshold, with Asp and Val no longer meeting the criterion for decreased amino acids, while Glu no longer met the criterion for increased amino acids in human studies. For animal studies, Met, Val, and OH-Pro no longer met the criterion for classification as consistently decreased amino acids, while Gln no longer met the criterion for consistently increased amino acids. Across both study types, circulating Val showed one of the least robust directional-consistency patterns in the present review. These findings, particularly those concerning circulating Val, should therefore be interpreted with caution, as their directional-consistency classification was sensitive to the selected threshold.

A notable observation in blood-based studies is the heterogeneity of reported findings between animal and human studies. In addition to differences in the amino acids showing directional consistency within each study type, some amino acids exhibited trends toward opposite directions of change in animal and human studies (e.g., Gln, Gly, and Phe), although these differences did not meet the predefined 70% directional-consistency threshold. Overall, the observed discrepancies raise important questions regarding the extent to which animal models of depression accurately recapitulate the molecular alterations observed in patients. At least some of the variability within animal studies may stem from the inherent limitations of depression models, while human studies could be influenced by unrecognized comorbidities. Moreover, recent work has highlighted the potential impact of chirality on amino acid measurements, which may further contribute to heterogeneity in the literature [26].

The available evidence suggests a predominant reduction in circulating amino acid levels in depressive conditions. The overlapping findings align with the monoamine hypothesis of depression, as both Trp and Tyr, key precursors for serotonin and catecholamine synthesis, are consistently reported as decreased. In addition, consistent reductions in Met may both contribute to and result from dysregulated neurotransmitter systems. Reduced Met availability could indirectly affect neurotransmitter synthesis through decreased production of its downstream metabolite, S-adenosylmethionine (SAM), a universal methyl donor essential for neurotransmitter metabolism. Importantly, the relationship between dietary Met intake and SAM levels in the central nervous system (CNS) has been previously documented [27].

**Figure 5:**
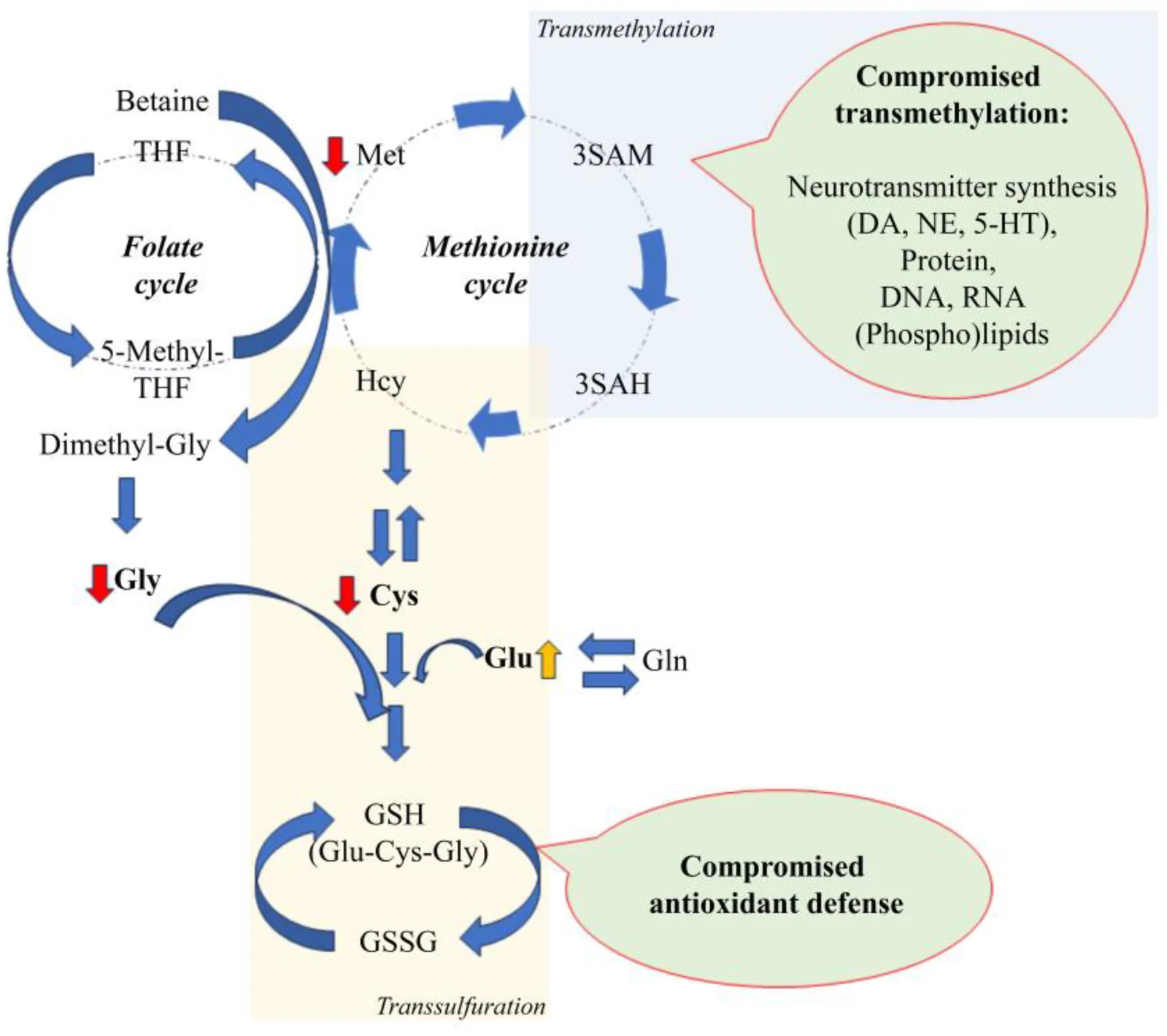
Schematic representation of one-carbon metabolism and its links to neurotransmitter synthesis and glutathione-dependent antioxidant defense. Arrows indicate the observed direction of change in specific amino acids (↑ increased; ↓ decreased), based on a 70% directional consistency threshold across non-targeted metabolomics studies. Key metabolites and cofactors are indicated: SAM—S-adenosylmethionine, SAH—S-adenosylhomocysteine, THF—tetrahydrofolate, DA—dopamine, NE—norepinephrine, 5-HT—serotonin, GSH—glutathione.

SAM participates in one-carbon metabolism, encompassing the folate and Met cycles (Figure 5). Folate deficiency has been observed in a subgroup of patients suffering from depression [28] and is correlated with deficits in CSF neurotransmitters, including dopamine, noradrenaline, and serotonin, in patients with depression [29]. Conversely, increased metabolic demand due to altered neurotransmitter turnover may impose constraints on SAM-dependent methylation processes [30], thereby contributing to systemic Met depletion. Beyond its role in monoamine synthesis, impaired methylation has been associated with major depression at multiple molecular levels [31], prompting both preclinical and clinical studies to explore SAM supplementation as a potential antidepressant strategy [32,33,34,35]. Nevertheless, the origin of reduced circulating Met remains unclear, and the functional consequences of its depletion, together with the potential benefits and risks of supplementation, require further investigation.

Alterations in Met metabolism may contribute to reduced Cys and Gly levels, alongside elevated Glu concentrations, all of which are recurrently observed in the human studies reviewed here. Both Glu and Cys represent key factors for glutathione synthesis [36]. Collectively, these changes may reflect a coordinated metabolic shift that compromises glutathione synthesis and, consequently, glutathione-dependent antioxidant defenses in depression (Figure 5). However, current evidence on blood glutathione-mediated antioxidant function in depressive states remains limited and variable, as most studies have focused on enzymatic activity rather than direct measurements of glutathione metabolites. This underscores the need for integrated analyses combining metabolite levels with enzymatic activity across distinct depressive phenotypes [37].

Beyond monoamine-related mechanisms, Leu and Val displayed consistent alterations across both human and animal studies, highlighting a potential role for branched-chain amino acid (BCAA) dysregulation in depressive and depressive-like phenotypes.

In non-targeted metabolomic studies, human investigations more frequently report increased circulating Glu, whereas animal studies often identify Gln as the altered metabolite. Given the rapid interconversion of these amino acids, such findings likely reflect species- and model-specific regulation of the Glu-Gln metabolic axis, rather than opposing biological effects. Elevated Glu in human studies and increased Gln in animal models may therefore represent distinct steady-state manifestations of dysregulated Glu-Gln metabolism, shaped by species-specific buffering capacity, disease chronicity, and methodological constraints inherent to non-targeted metabolomics.

In parallel, consistent reports of elevated circulating Glu and reduced GABA levels in human studies point to a systemic excitatory–inhibitory imbalance. Although peripheral metabolite levels do not directly reflect synaptic neurotransmission, these alterations may indirectly relate to central glutamatergic dysfunction, aligning with hypotheses implicating glutamate-mediated excitotoxic mechanisms in depression. Nevertheless, the limited number of studies combining peripheral and CNS samples precludes firm conclusions regarding the extent to which circulating Glu or GABA accurately reflect brain excitatory–inhibitory balance.

Some amino acid derivatives, such as N-acetylated forms of Asp, Glu, and Gln, have been reported primarily in animal studies. These changes generally parallel alterations in their corresponding free amino acids and may reflect temporary nitrogen buffering or excitatory amino acid storage mechanisms. Notably, N-acetyl aspartate (NAA), as one of the most abundant metabolites in the CNS, is considered a predominantly neuronal metabolite and is widely used as a marker of neuronal integrity [38]. The elevated blood levels could indicate spillover from the CNS or altered brain–periphery compartmentalization. However, recent research has identified brown adipose tissue as a significant source of NAA and has highlighted its role in energy metabolism and metabolic adaptations within brown adipose tissue [39]. Other derivatives, such as N-Ac-Trp and N-Ac-Tyr, were reported only once and may reflect depletion driven by increased brain demand. These observations remain preliminary and warrant cautious interpretation.

### 5.2. Amino Acid Dysregulation in CNS Matrices

Study-level findings on amino acid alterations reported in CNS matrices are summarized in Table S4, while the results of the vote-counting analysis are presented in Table 2. Substantial heterogeneity in amino acid dysregulation is evident across different CNS matrices; however, inconsistencies appear less pronounced than in blood-based studies. It should be noted that all CNS-related studies were conducted exclusively in animal models, thereby avoiding potential confounding factors specific to the human brain and eliminating one source of heterogeneity present in peripheral analyses.

**Figure 6:**
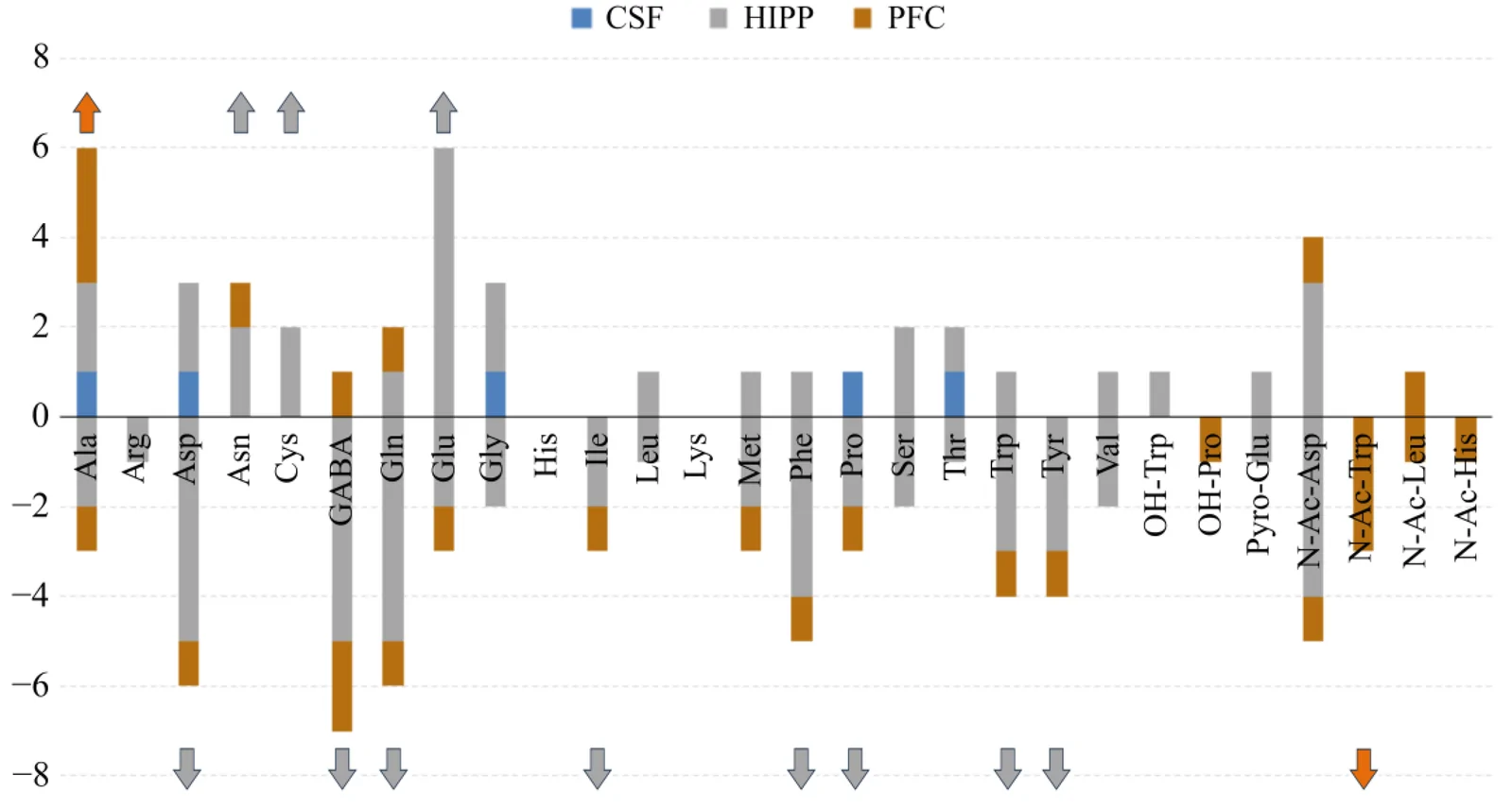
Graphical representation of amino acid and derivative alterations in CNS samples. Bars indicate the number of independent non-targeted metabolomic studies reporting significantly upregulated (above zero) and downregulated (below zero) levels for each metabolite in animal models of depression, based on CSF, HIPP, and PFC samples. Arrows denote metabolites exhibiting directional consistency among significant reported findings (≥70%) across ≥2 independent studies, presented separately for each brain region.

Figure 6 illustrates the results of the vote-counting analysis separately for each CNS matrix, using the primary 70% directional-consistency threshold. The robustness of these classifications across alternative thresholds (60% and 80%) is presented in the sensitivity analysis (Table S5). Brain regions such as the HIPP and PFC are critical for emotional regulation, memory, learning, and executive function, all of which are impaired in depression. The HIPP, in particular, exhibits volume loss correlated with depression severity, along with impaired neurogenesis and synaptic dysfunction [40,41]. Dysregulation of HIPP-PFC circuits has been widely reported in depression, with altered connectivity contributing to cognitive and affective deficits [42]. In the HIPP, non-targeted metabolomics studies have consistently reported decreases in Asp, GABA, Gln, Ile, Phe, Pro, Trp, and Tyr, alongside increases in Asn, Cys, and Glu, based on statistically significant reported findings. Compared to blood-based studies, greater unambiguous directional consistency is observed for several amino acids, including elevated Asn and Cys and decreased GABA, Ile, Pro, and Tyr.

The sensitivity analysis (Table S5) showed that several HIPP findings remained consistent across all three thresholds (60%, 70%, and 80%), including increases in Asn and Cys and decreases in GABA, Gln, Ile, Phe, Pro, and Tyr. In contrast, the classifications of Asp, Glu, Met, Trp, and Val varied with the selected threshold.

Continuing the patterns observed in blood, reduced levels of Trp and Tyr were frequently reported in non-targeted metabolomic studies of the HIPP (Figure 6). This blood–brain association is particularly compelling for Trp, an essential amino acid that enters the brain via peripheral supply through the large neutral amino acid transporter 1 (LAT1) (see next subsection), strengthening the biological plausibility of a link between peripheral and central Trp depletion. Indeed, a few translational studies of depression have reported concurrent reductions in circulating and hippocampal Trp levels [43,44]. Hence, the reduction in essential amino acids such as Trp, Phe, and Ile in the HIPP may reflect reduced transport across the BBB due to either decreased circulating levels or impaired transporter function, increased utilization within relevant biochemical pathways, or a combination of these mechanisms. Additionally, there is evidence suggesting impaired functionality of BBB transporters, which will be discussed in more detail in the following subsection.

Early evidence has linked depression to alterations in inhibitory circuits, mediated primarily by GABA, the most abundant inhibitory neurotransmitter. Non-targeted metabolomic analyses have consistently reported declines in both circulating and hippocampal GABA levels. This reduction has been observed in both depressed patients and animal models of depression, particularly within the HIPP. Supporting the functional relevance of GABA in depression, effective antidepressant treatments have been shown to normalize hippocampal GABA levels [45,46]. Corroborative evidence also comes from novel psychobiotic approaches, in which GABA-producing microbiota show promise in preventing severe depression in people with subthreshold depression and alleviating depressive symptoms in animal models of depression [47,48]. In parallel, the consistent reports of elevated Glu and decreased Gln highlight the importance of pathophysiological changes in the excitatory–inhibitory axis of the HIPP. Supporting this notion, Yu et al. reported reduced GABA levels in both plasma and HIPP of rats subjected to chronic unpredictable mild stress (CUMS) [43], whereas Zhu et al. observed increased Glu levels in both serum and HIPP in the same model [44]. The concurrent decrease in Asp and increase in Asn, together with elevated Glu and reduced Gln levels in the HIPP, suggests a potential compensatory redistribution of nitrogen. The enhanced conversion of Asp to Asn may represent a protective nitrogen-buffering mechanism under conditions of disrupted Glu-Gln metabolism and excitatory stress, potentially involving altered astrocytic function. However, given the steady-state nature of non-targeted metabolomics data, this interpretation remains speculative and warrants further investigation. Alternatively, these compartment-specific amino acid alterations may reflect intrinsic cerebral metabolic dysregulation, including changes in astrocyte–neuron metabolic coupling and local amino acid utilization. Such mechanisms are supported by evidence of altered GABAergic function in depression, including changes in the expression of GABA-synthesizing enzymes such as GAD67 and dysfunction of parvalbumin-positive interneurons, although the findings vary across brain regions and study models.

Interestingly, a remarkable contrast emerges for Cys, with peripheral levels consistently decreased among significant findings, yet hippocampal levels consistently increased. Notably, Cys in the brain is not solely dependent on blood levels. Neurons and glia can synthesize Cys de novo via the transsulfuration pathway from Met, while uptake from blood occurs primarily in the form of cystine through the system Xc- antiporter, in exchange for Glu, followed by intracellular reduction to Cys in astrocytes [36]. Since Cys availability is crucial and rate-limiting for glutathione synthesis, the regional elevation of hippocampal Cys may therefore reflect a compensatory adaptation to excitatory or oxidative stress, supporting both glutathione-dependent and glutathione-independent antioxidant defense (through its reactive thiol group), rather than a direct mirror of peripheral Cys levels [49]. In addition, cystine transport at the level of Xc- antiporter has been shown to be sensitive to inhibitory effects of increased extracellular Glu levels in the brain; that is, higher extracellular Glu levels inhibit cystine uptake by astrocytes in exchange for Glu release from astrocytes, linking brain cystine levels to the Glu excitatory theory of depression [50]. However, currently the available results derived from non-targeted studies are scarce and insufficient to draw conclusions regarding regional Cys abundance in the brain.

CSF provides mechanical protection, facilitates nutrient delivery and waste clearance, supports immune signaling, and distributes neuroactive molecules, thereby maintaining brain homeostasis [51]. The choroid plexus–CSF axis acts as a dynamic regulator of the brain’s extracellular environment [52]. Consequently, CSF composition likely reflects CNS biochemical states, and alterations in CSF metabolites can indicate neuroinflammation, neurodegeneration, or other pathological processes [53,54,55]. Under the applied inclusion criteria, only one non-targeted CSF metabolomics study was identified, reporting increased levels of Ala, Asp, Gly, Pro, and Thr. Consequently, the biological relevance of these findings is limited, highlighting the need for additional non-targeted metabolomic studies in CSF. A major challenge is the invasiveness of CSF collection in clinical settings, together with the limited sample volumes obtainable from animal models, which can constrain comprehensive non-targeted analyses [56]. Nevertheless, targeted CSF metabolomics studies have identified depression-associated metabolic abnormalities, supporting the potential value of CSF molecular profiling for biomarker discovery [57]. Some studies have also reported significant correlations between metabolite changes in CSF and serum, suggesting that serum may, in certain contexts, serve as an informative surrogate for CSF. A recent study investigating causal relationships between CSF metabolites and depression risk identified 64 metabolites with potential involvement [58].

Similar to CSF, only a limited number of non-targeted metabolomic studies have reported significant amino acid dysregulations in the PFC. Among the reported findings, Ala was increased and N-Ac-Trp decreased at the 70% directional-consistency threshold, whereas only the decrease in N-Ac-Trp remained consistent at the 80% threshold (Figure 6; Table S5). Notably, the reported decrease in N-Ac-Trp showed 100% directional consistency, although this finding should be interpreted cautiously given the limited number of available studies. Further non-targeted metabolomic studies are needed to establish more robust amino acid profiles in both CSF and the PFC.

## 6. CNS–Blood Metabolite Communication—Brain Barrier Systems

To understand the systemic nature of depression and to drive progress in diagnostics and therapy monitoring, it is essential to determine whether metabolic alterations detected in peripheral biofluids reflect those occurring in specific brain regions, or conversely whether central metabolic changes can be detected in peripheral compartments. However, most metabolomics studies have focused on either peripheral or central samples, limiting our understanding of metabolite dysregulation across the brain–body axis.

### 6.1. Brain Barriers and Amino Acid Compartmentalization

Metabolite exchange between peripheral and central compartments is regulated by three major brain interfaces: the BBB, blood–CSF barrier, and CSF–brain interface. Together, they maintain CNS homeostasis through selective transport rather than passive diffusion and are dynamically altered by inflammation, chronic stress, and endocrine disturbances implicated in depression [59]. Impaired barrier integrity has been demonstrated in animal models of depression [60], while neurovascular dysfunction and increased BBB permeability have also been reported in patients with depression [61,62].

Among these barriers, the BBB is the most restrictive, with molecular exchange occurring predominantly through specialized transporters [63,64]. The blood–CSF barrier is comparatively more permeable but remains highly selective owing to epithelial transport mechanisms [65]. Consequently, proteinogenic amino acid concentrations in CSF are generally 2- to 20-fold lower than in blood, with glutamine representing a notable exception [66]. In contrast, the CSF–brain interface permits relatively free exchange of small hydrophilic molecules between CSF and the brain parenchyma [67], and recent evidence suggests that ependymal cell function at this interface contributes to depression-related behaviors [68]. Detailed reviews of the morphology and physiology of these barrier systems are available elsewhere [69].

### 6.2. Transporter Systems Regulating CNS Amino Acid Availability

Amino acid exchange across CNS barriers is tightly regulated by selective transporters operating at the BBB, blood–CSF barrier, and CSF–brain interface. These interfaces not only maintain CNS homeostasis but also determine the central availability of both essential and non-essential amino acids. Non-essential amino acids are synthesized endogenously and contribute substantially to circulating and tissue amino acid pools. In contrast, essential amino acids (Trp, Phe, Ile, Leu, Lys, Val, Met, Thr, and His) cannot be synthesized de novo in humans and therefore depend on dietary intake and protein turnover. Their CNS availability is determined by specialized transport systems located at brain barrier interfaces.

Multiple transporter families mediate amino acid exchange across brain barriers, with substrate specificity determining the availability of essential amino acids, neurotransmitter precursors, and metabolic intermediates within the CNS (Table 3). Among BBB transport systems, LAT1 is particularly relevant because it mediates the transport of several large neutral amino acids, including Trp, Tyr, Phe, Met, and BCAAs, which are frequently altered in depression metabolomics studies [64,70,71,72]. Besides LAT1, BBB endothelial cells express additional transporter systems, including cationic amino acid transporter (CAT), system X^−^c, y+LAT2, and SNAT1/2 [70]. Evidence for BBB localization of ASC is limited. In contrast, EAAT1–3 are primarily expressed in astrocytes and neurons, where they contribute to the regulation of extracellular acidic amino acid levels. EAAT expression in astrocytic endfeet adjacent to blood vessels has also been reported [73]. Together, these transport systems contribute to the transport and regulation of cationic, neutral, and acidic amino acids.

The choroid plexus represents an additional regulatory interface controlling CSF amino acid composition [74]. In addition, new studies highlight the importance of the choroid plexus in regulating brain inflammation through its interactions with immune cells, as it represents an immune cell hub [75]. Compared with the BBB, it exhibits a distinct transporter repertoire, including LAT2, SNAT1/3, ASC1, and SIT1, together with several additional transport systems (Table 3) [66,72]. Other transporters, such as the sodium-dependent B^0^ amino acid transporter (B^0^AT2), are also present at the choroid plexus but appear to have a more limited distribution. B^0^AT2 is predominantly localized to neurons and astrocytes [76,77]. However, the contribution of choroid plexus transporter alterations to depression-associated amino acid changes remains poorly characterized. In addition, SNAT1 was reported to be the crucial transporter involved in the Gln influx from the blood into epithelial cells, while LAT2 and SNAT1 mediate Gln export from epithelial cells into the CSF at the abluminal side [66].

Most of these transporters are saturable, while competitive transport is particularly characteristic of Na^+^-independent transporters such as LAT1 [64]. LAT1 mediates passive, yet competitive diffusion at both the luminal and abluminal sides of the BBB, providing a mechanism linking systemic amino acid availability to central pools. Furthermore, all amino acid exchange is gradient-coupled and hence tightly regulated and dependent on the ongoing metabolic processes. This transport selectivity provides a mechanistic framework for interpreting the compartment-specific amino acid alterations identified in the present metabolomic analysis.

**Table 3:** Tabular representation of amino acid transporters expressed at the level of brain barriers and interfaces.

Transport System	Transporter (Gene)	Amino Acids Transported	Typical Localization
Na^+^-independent transporters			
System L (Antiport) [66,78]	LAT1 (*slc7a5 + slc3a2*)	Trp, Tyr, Phe, Met, His, Thr, Leu, Ile, Val	BBB (high), astrocytes, neurons
	LAT2 (*slc7a8*)	Neutral AA exchange	choroid plexus
System Y^+^ (Uniport-facilitated diffusion) [79,80]	CAT (*slc7a1-a3*)	Arg, Lys	BBB, choroid plexus
System N (Symport/antiport) [66]	SNAT 3/5 (*slc38a3/a5*)	Gln-Glu cycling	choroid plexus
System ASC (Antiport) [81,82,83]	ASCT1/2 (*slc1a4/slc1a5*)	Ala, Ser, Cys	choroid plexus, BBB
System X^−^c (Antiport) [66]	X^−^c (*slc7a11* (+*slc3a2*))	Cystine/Glu exchange	BBB, choroid plexus (moderate)
Na^+^-dependent transporters			
System A (Na^+^ + AA symport) [66,81]	SNAT 1/2 (*slc38a1/a2*)	Small neutral AAs; Gln supply	choroid plexus (high), BBB (moderate)
System Y^+^L (Antiport—cationic ↔ neutral + Na^+^) [66]	y^+^LAT2 *slc7a6*/*slc7a7* (+*slc3a2*)	Cationic and neutral amino acids—Arg/Lys	BBB and choroid plexus
Symport—Na^+^ + Pro [66,84]	SIT1 (*slc6a20*)	Pro, Gly	choroid plexus
Transporters mainly localized in neural cells			
System B^0^ [76]	B^0^AT2 (*slc6a15*)	Neutral AA (BCAAs, Pro, Met)	Neurons mainly, astrocytes, choroid plexus
System X^−^_AG_ (Symport—Na^+^ + Glu) [73]	EAAT1-3 (*slc1a1-3* family)	Glu	Astrocytes and neurons, minimal at BBB

Taken together, the coordinated activity of these transporter systems maintains amino acid compartmentalization between systemic circulation and the CNS. Altered transporter expression or function may disturb this balance, modify the central availability of essential amino acids, and consequently contribute to metabolic and neurotransmitter abnormalities implicated in depression.

### 6.3. Barrier Dysfunction in Depression

Accumulating evidence indicates that the integrity of multiple brain barrier systems is compromised in depression. A significant positive association has been reported between depression severity and BBB leakage in the HIPP and thalamus [62]. Moreover, BBB properties exhibit pronounced regional heterogeneity, and sex-specific vulnerability to stress-induced depression has been linked to region-dependent BBB dysfunction, particularly within the PFC and limbic structures [61].

The involvement of BBB pathology in depression has been proposed based on findings of altered distribution of specific molecules, most notably S100B, an astrocyte-derived protein, as well as other CNS biomarkers detected in the blood and CSF of depressed patients [85]. Multiple mechanisms have been implicated in BBB dysfunction in depression. At the molecular level, proinflammatory cytokines such as IL-6, IL-1β, and TNF-α have been shown to impair the integrity of both the BBB and the blood–CSF barrier, thereby facilitating atypical peripheral-to-CNS molecular exchange [86,87]. In line with this, growing evidence supports the concept of CSF as a sensor and transducer of peripheral metabolic and inflammatory states to the CNS [88]. Experimental studies demonstrate that chronic stress can reduce BBB integrity through downregulation of tight-junction proteins, including the endothelial-specific protein claudin-5, enabling peripheral immune signaling and contributing to depressive-like phenotypes [89]. In addition, antidepressant treatments have been shown to restore tight-junction proteins [90]. Beyond the cerebral vasculature, peripheral endothelial dysfunction has also been associated with depressive symptomatology, supporting the concept that endothelial cells actively contribute to, rather than merely reflect, depression pathophysiology [91,92].

An early study demonstrated that the choroid plexus responds to chronic stress in an animal model of depression, identifying changes in the expression of several genes and providing some of the first evidence linking choroid plexus alterations to depression-related phenotypes [93]. Disruption of the choroid plexus transcriptome in depression was also confirmed in a post-mortem study [94]. More recent imaging studies indicate that choroid plexus enlargement is associated with brain inflammation, depression severity, illness duration, systemic oxidative stress, and peripheral inflammatory markers [95,96,97,98]. The novel view of the choroid plexus as a specialized organ orchestrating the immune defense through the coordinated influx of immune cells has gained increasing attention, highlighting the importance of this structure under inflammatory brain conditions [75]. In addition, reductions in choroid plexus and brain ventricle volumes have been proposed as predictors of antidepressant treatment response in patients [96], while increased choroid plexus volumes have also been associated with treatment-resistant depression [99]. Collectively, these findings position the choroid plexus as a dynamic neuroimmune interface whose dysfunction may contribute to altered metabolite exchange between peripheral circulation and the CNS in depression.

### 6.4. Evidence for Depression-Associated Alterations in Amino Acid Transport Regulation

In addition to structural alterations, barrier dysfunction may also result from changes in transporter expression and activity. Such changes may occur in brain endothelial cells, choroid plexus epithelial cells, and their associated transport systems, including LAT, SNAT, ASC, CAT, and EAAT transporter families, which regulate amino acid exchange between blood and brain [100]. Amino acid transporter dysregulation has already been reported in several neurological disorders [101]. Although several studies have also reported alterations in mitochondrial and vesicular amino acid transporters in depression and following antidepressant treatment [102,103,104], these systems primarily regulate intracellular metabolite handling or synaptic neurotransmitter storage. Because they do not directly mediate amino acid exchange across brain barrier interfaces, they are not discussed in detail in the present review.

**Figure 7:**
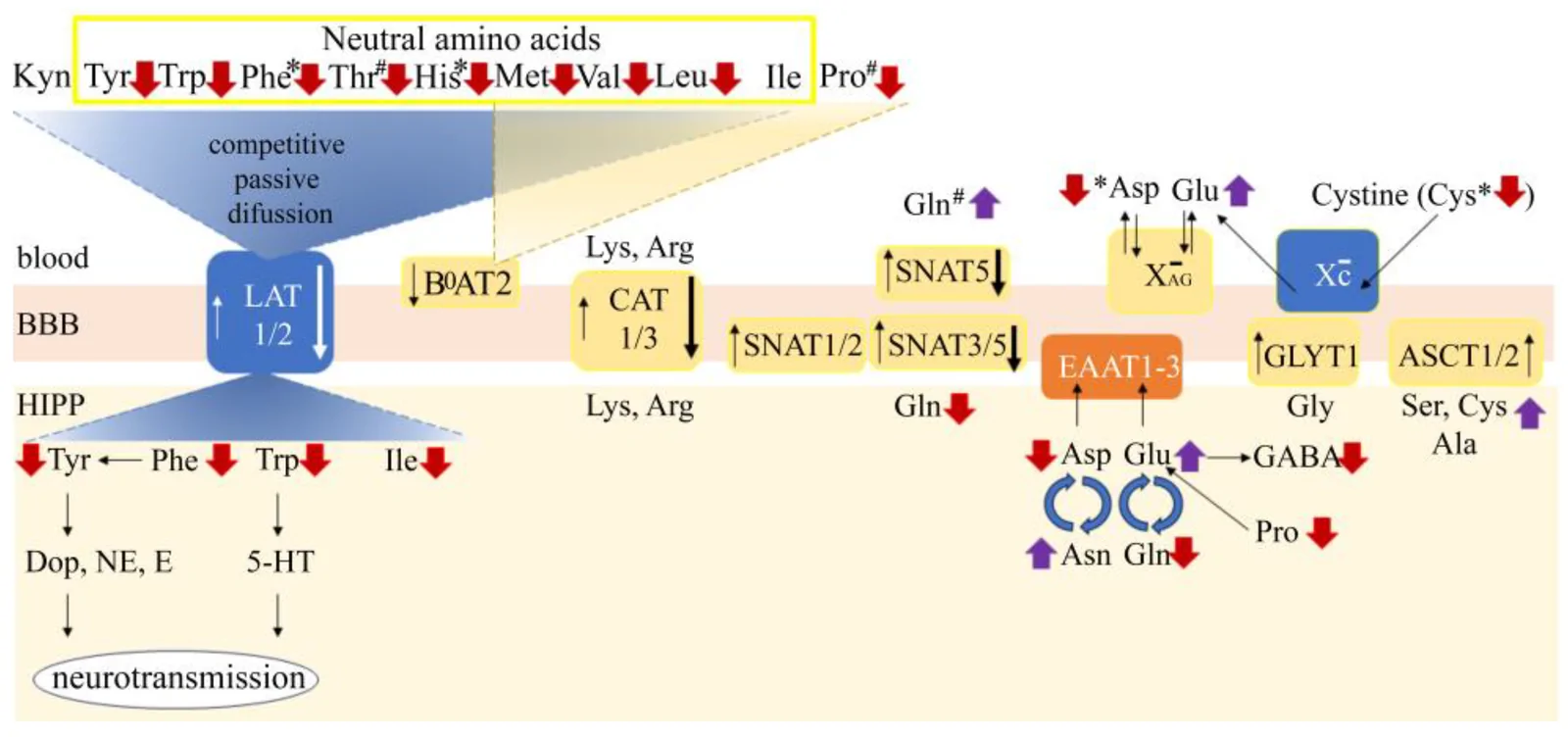
Schematic representation of amino acid transport across the BBB with individual amino acid dysregulations in depression depicted. Arrow directions indicate the directionality of reported amino acid alterations, with a 70% threshold applied to define consistent changes. Asterisks (*) denote metabolites for which changes have been reported only in human studies, whereas hash symbols (#) indicate alterations observed exclusively in animal studies.

Interpretation of transporter alterations is complicated by the fact that most amino acid transporters are expressed in multiple CNS cell types. Consequently, available studies generally assess transporter expression in whole-brain tissue or neuronal and astrocytic populations rather than isolated BBB endothelial or choroid plexus epithelial cells. Direct evidence for barrier-specific transporter alterations in depression therefore remains scarce and represents an important knowledge gap. Figure 7 integrates transporter specificity with the amino acid alterations identified as consistent in the present vote-counting analysis. Among the transport systems, LAT1 is particularly relevant because it mediates CNS uptake of several amino acids that were consistently decreased in both circulation and HIPP. Acute Trp depletion studies provide strong evidence that reduced circulating Trp levels influence the same neural pathways affected by acute mental stress [105]. In addition, a meta-analysis found that Trp levels were negatively correlated with depression rating scores [13]. Brain Trp availability depends on its transport from the circulation, and reductions may arise from low circulating levels, impaired transport, increased central utilization, or all three. Importantly, dietary Trp intake per se has not been consistently linked to depression onset, but rather to changes in serotonergic vulnerability that predispose certain individuals to depressive states [106]. Beyond serotonin synthesis, Trp serves as a precursor for multiple metabolic pathways, with the majority entering the kynurenine pathway [107]. Dysregulation of this pathway, particularly imbalances between kynurenic and quinolinic acid, has emerged as a prominent feature in depression research and is among the most frequently reported alterations in blood-based metabolomic studies. In line with this concept, Trp supplementation has been explored as both a therapeutic and prophylactic strategy for depressive disorders [108].

Although transporter dysfunction represents a plausible mechanism linking peripheral and central amino acid alterations, current evidence remains largely indirect because metabolomic studies and transporter studies have rarely been performed within the same experimental cohorts.

#### 6.4.1. Large Neutral Amino Acid Transporter (LAT)

LAT1 (slc7a5) is the predominant transporter mediating BBB uptake of large neutral amino acids, including Trp, Tyr, Met and BCAAs, making it particularly relevant to the metabolomic alterations identified in the present review [64,72].

Inflammation represents one of the core mechanisms explored and reported in a subset of depression phenotypes. Wittmann and colleagues reported that lipopolysaccharide-induced inflammation reduces LAT1 mRNA levels in brain blood vessels of both male Sprague-Dawley rats and C57Bl/6 mice [109]. On the other hand, no changes in LAT1 and LAT2 mRNA expression were observed in neurons or choroid plexus epithelial cells. In addition, dysregulated LAT1 expression has also been linked to environmentally induced depressive phenotypes [110].

Altered LAT1-mediated transport has been proposed as a mechanism contributing to antidepressant effects. Specifically, the antidepressant effects of Leu supplementation have been demonstrated in LPS-induced depression-like behavior, in which Leu was shown to modulate LAT1-dependent transport processes [111]. LAT1 is also a primary transporter for kynurenine, a key intermediate of Trp metabolism implicated in neuroinflammation and excitotoxicity. Leu has been proposed to compete with kynurenine, limiting its brain accumulation and mitigating downstream neurotoxic metabolite formation in inflammation-driven depression [111]. In this model, antidepressant effects were observed in parallel with increased LAT1 expression mediated via the mTOR signaling pathway, whereas depressive-like behavior was associated with reduced LAT1 expression [112]. These findings suggest that both transporter expression levels and substrate availability may critically influence the brain uptake of large neutral amino acids and, consequently, CNS exposure to their neuroactive metabolites. Accordingly, the consistent reduction in circulating Leu levels observed across both human and animal studies raises the possibility that impaired amino acid transport in depression may arise not only from altered LAT1 expression but also from limited substrate availability. Moreover, competitive inhibition at LAT1 is unlikely to selectively affect kynurenine alone. Rather, it may broadly influence the transport of other essential amino acids sharing this carrier system. Consequently, imbalances in peripheral amino acid pools may propagate to the CNS and influence central metabolic and neurotransmitter homeostasis. Building on this principle, LAT1 has been proposed as a potential therapeutic target in depression, particularly in the context of inflammation-associated phenotypes.

#### 6.4.2. B^0^AT2

B^0^AT2 (slc6a15) is expressed predominantly in neurons and astrocytes, with additional expression in choroid plexus epithelial cells, and is particularly abundant in the HIPP, cerebral cortex, hypothalamus, and olfactory bulb [76]. Unlike BBB transporters, B^0^AT2 does not directly mediate amino acid exchange between the circulation and the CNS, but regulates intracellular uptake of neutral amino acids, including BCAAs, Pro, and Met, thereby contributing to local amino acid homeostasis [113].

Genetic and experimental studies consistently implicate slc6a15 in depression susceptibility. Polymorphisms and reduced expression of the slc6a15 gene have been linked to stress vulnerability [114,115], alterations in resting-state activity across several brain regions, HIPP atrophy, changes in adrenocorticotropic hormone and cortisol secretion, cognitive impairments in unipolar depression, and reduced white matter integrity in patients with major depressive disorder (MDD) [116,117,118]. Functional studies demonstrate that slc6a15 modulates anxiety- and depressive-like behaviors, likely through effects on glutamatergic neurotransmission. Santarelli and colleagues reported that experimental increases in slc6a15 expression in the HIPP induce behavioral and molecular alterations comparable to those observed following chronic stress exposure. They further demonstrated that slc6a15 levels modulate hypothalamic–pituitary–adrenal axis activity as well as Glu receptor expression in the HIPP. Accordingly, Santarelli et al. identified slc6a15 as a “crucial factor controlling amino acid content in the hippocampus” [119,120].

Experimental evidence further indicates that slc6a15 expression is dynamically regulated by stress and antidepressant interventions. Chronic stress, corticosterone exposure, and the chronic social defeat stress (CSDS) model are generally associated with reduced slc6a15 expression, whereas several antidepressant-like interventions restore or increase its expression, although not all pharmacological studies report consistent findings [116,121,122,123].

Mechanistically, reduced B^0^AT2 activity has been proposed to decrease Pro availability and disrupt glutamatergic neurotransmission, contributing to elevated hippocampal Glu/Gln ratios [119]. Consistent with the metabolomic findings of the present review, alterations in Pro, Leu, and related neutral amino acids support a potential contribution of B^0^AT2 dysfunction to depression-associated metabolic abnormalities [76]. Consequently, B^0^AT2 has emerged as a promising therapeutic target for psychiatric disorders, including depression [77,124,125].

#### 6.4.3. ASC Transport System

ASC transporters (ASCT1/slc1a4 and ASCT2/slc1a5) regulate intracellular exchange of small neutral amino acids, including Ala, Ser and Cys, and are predominantly expressed in neurons and astrocytes. Although endothelial expression has also been reported, available depression studies primarily examine neuronal and astrocytic expression rather than transport across brain barriers, leaving the contribution of endothelial ASC transport largely unexplored.

Available evidence suggests differential regulation of ASC transporters in depression. Increased slc1a5 (ASCT2) expression has been associated with high-risk MDD [126] and was also reported in the CSDS model, where its downregulation alleviated depressive-like behavior [127]. In contrast, hippocampal slc1a4 (ASCT1) expression was reduced in patients with depression [128], indicating transporter-specific rather than uniform alterations within the ASC system.

Consistent with the vote-counting analysis presented in this review, non-targeted human studies consistently report reduced circulating Ala levels in depression. Supporting a functional role of the ASC system, alanine administration produced antidepressant-like effects while reducing hippocampal ASCT1 and ASCT2 expression without affecting the PFC [129]. Likewise, pharmacological inhibition of ASCT1/2 with L-4-fluorophenylglycine attenuated depressive-like behavior in the CSDS model, potentially by limiting D-Ser efflux from the brain [130]. Although the underlying mechanisms remain incompletely understood, the available evidence suggests that ASC transporters contribute to amino acid homeostasis relevant to depression pathophysiology. Nevertheless, whether these alterations reflect impaired amino acid transport across brain barrier interfaces or cell-specific metabolic adaptations remains unresolved.

#### 6.4.4. Other Amino Acid Transporters Implicated in Depression

Beyond endothelial transport systems, astrocytes also contribute to CNS amino acid homeostasis by regulating extracellular amino acid concentrations and maintaining BBB integrity through their perivascular end-feet [131,132,133]. Astrocytic dysfunction has been implicated in the metabolic and neuroinflammatory alterations associated with depression, and reduced expression of astrocytic glutamate transporters may further disrupt amino acid homeostasis. EAAT 1-4 represent the most extensively studied amino acid transporters and belong to the slc superfamily of transporters. Human postmortem studies report a decline in astrocytic EAAT2 expression in the olfactory bulbs [134], as well as altered expression of hippocampal SLC1A2 (EAAT2) and SLC1A3 (EAAT1) in depression [102]. Animal studies also report hippocampal EAAT2 downregulation in stress-maladaptive rats, while no changes were observed in EAAT1, EAAT3 and EAAT4 expression [135]. Antidepressant effects have been associated with both EAAT1 downregulation [136] and upregulation [137] in the PFC. Although altered serum and CSF levels of the astrocytic marker S100B have been reported in depression, their relationship with BBB permeability remains uncertain [138,139,140]. Nevertheless, because amino acid exchange between blood and brain is primarily mediated by endothelial transporters, astrocytic dysfunction is likely to influence depression-associated amino acid alterations indirectly through modulation of the extracellular metabolic environment rather than trans-barrier transport.

Altered amino acid transporter expression has also been reported in the intestinal epithelial cells. Chronic stress models, including CUMS and maternal separation, induce LAT1 upregulation together with ASCT2 and B^0^AT1 downregulation, suggesting that impaired intestinal amino acid absorption may contribute to the reduced circulating Gly and Gln levels observed in depression [141,142].

Collectively, the available evidence identifies LAT1 and B^0^AT2 as the amino acid transporters with the strongest mechanistic support in depression, whereas evidence for other transporter systems remains limited and is derived predominantly from whole-brain or mixed-cell analyses rather than brain barrier-specific investigations. This highlights the need for cell-specific studies integrating transporter expression with metabolomic profiling to determine whether altered amino acid transport directly contributes to the compartment-specific metabolic signatures observed in depression. Although transporter dysregulation has been extensively explored in other neurological disorders, including Alzheimer’s disease [143], it remains comparatively underexplored in depression. The available findings support the concept that barrier-regulated amino acid transport represents an important mechanistic link between peripheral metabolic disturbances and central dysfunction in depression, while emphasizing the need for studies specifically addressing the BBB, blood–CSF barrier, and CSF–brain interface. Overall, integrating transporter biology with non-targeted metabolomic profiling provides a mechanistic framework for interpreting compartment-specific amino acid dysregulation in depression and may facilitate the identification of more biologically informed biomarkers and therapeutic targets.

## 7. Methodological Considerations and Limitations

The findings of this review should be interpreted in light of several limitations. First, substantial heterogeneity in biological matrices, analytical platforms, metabolite annotation, and study populations contributes to variability across metabolomic studies and may partly explain inconsistencies in reported amino acid alterations. Second, the vote-counting approach used in the present review identifies the consistency and direction of metabolite changes across significantly reported findings but does not account for effect sizes or study quality. The sensitivity analysis showed that the classification of some amino acids depended on the selected directional-consistency threshold. Although several amino acids retained the same directional classification across the tested thresholds, these results should be interpreted in the context of the vote-counting approach. Because only statistically significant reported changes were systematically extracted, the calculated percentages reflect directional agreement among significant reported findings rather than among all studies measuring each amino acid. The included studies used heterogeneous criteria to identify and report differential metabolites, involving *p*-value or FDR thresholds, VIP scores, fold-change cutoffs, and metabolite identification or annotation criteria. However, the published reports did not consistently provide sufficient information to determine which metabolites were measured and evaluated but not reported, or why particular metabolites were absent from the reported results. Consequently, we could not systematically distinguish genuine null findings from metabolites that were not detected, identified, retained after analytical filtering, or reported. The absence of a formal risk-of-bias assessment and the inability to determine the reasons for unreported metabolite findings limit the evaluation of potential selective reporting bias. Therefore, the vote-counting results should be interpreted cautiously. Finally, depression is a clinically heterogeneous disorder, and factors including subtype, disease severity, medication status, sex, and age may influence metabolomic profiles but could not be systematically addressed within the available literature. Despite these limitations, integrating automated literature mining with systematic vote counting enabled the identification of amino acids showing consistent directional alterations across the included studies and biological compartments, providing a basis for future mechanistic and translational research. By considering the findings alongside current knowledge of brain barrier biology and amino acid transport in depression, this review also provides a framework for interpreting compartment-specific metabolomic alterations and identifying priorities for future experimental research.

## 8. Conclusions and Future Directions

Non-targeted metabolomics has consistently demonstrated that depression is accompanied by widespread metabolic alterations across both peripheral and central compartments. By integrating automated literature mining with systematic vote-counting analysis, this review identifies proteinogenic amino acids as the metabolite class most frequently reported as dysregulated across non-targeted metabolomic studies of depression. At the 70% directional-consistency threshold, human studies show a high degree of agreement in reporting reduced circulating levels of Trp, Tyr, Phe, Met, Leu, Val, Ala, Gly, Asp, Cys, His, Lys, and GABA. Most of these findings remain consistent at the 80% threshold, with only Val and Asp no longer meeting the criterion. Evidence for increased circulating amino acids is less robust, partly because of the limited number of studies. At the 80% threshold, only OH-Glu retains its classification as consistently increased, whereas Glu no longer meets the criterion. It is noteworthy that, despite limited concordance between preclinical and clinical studies, several circulating amino acids, including Trp, Tyr, Met, Leu, and Val, showed directionally concordant changes in human and animal serum samples at the 70% consistency threshold, supporting the potential translational relevance of animal studies. In the HIPP, the most consistent alterations included reduced GABA, Gln, Trp, Tyr, Ile, and Pro, together with increased Glu, Asn, and Cys, suggesting disruption of excitatory–inhibitory neurotransmission, amino acid homeostasis, and oxidative stress responses. In contrast, evidence from CSF and the PFC remains limited, indicating that additional non-targeted metabolomic studies are required to establish robust CNS amino acid signatures.

The observed patterns of essential amino acid alterations are biologically consistent with the substrate specificity of key amino acid transport systems, particularly LAT1 and B^0^AT2, which have been implicated in depression-related research. However, whether altered transporter activity contributes to the compartment-specific amino acid patterns observed in depression remains unclear. These patterns may reflect several non-exclusive mechanisms, including intrinsic metabolic changes within the brain, altered astrocyte–neuron metabolic coupling and local amino acid utilization, as well as potentially altered amino acid exchange across the BBB and blood–CSF barrier. This framework highlights brain barrier transport as one possible avenue for future research into the differences between peripheral and central metabolomic profiles in depression.

Future studies should integrate longitudinal metabolomics with clinical phenotyping, neuroimaging, and complementary multi-omics approaches, while incorporating measures of BBB and blood–CSF barrier function. Such integrative approaches may help distinguish trait from state metabolic alterations, clarify potential mechanisms, and facilitate the identification of biologically informed biomarkers and therapeutic targets. Future non-targeted metabolomic studies should also adopt more comprehensive reporting practices, making all detected and analyzed metabolite results available regardless of statistical significance or the specific filtering criteria applied. This would enable more complete and reliable comparisons across studies. Overall, this review summarizes the consistency of reported amino acid alterations in human and animal studies of depression across peripheral and CNS compartments and provides a translational framework for interpreting non-targeted metabolomic findings in the context of brain barrier transport biology. Future studies combining metabolomics with cell-specific analyses of amino acid transporter expression and function, alongside direct assessments of barrier function, are needed to determine whether altered transporter-mediated amino acid exchange contributes to differences between peripheral and central amino acid profiles in depression.

## Figures and Tables

**Table 1:** Vote-counting analysis of significantly increased and decreased circulating amino acids reported in animal and human studies. Directional consistency (%) was calculated for amino acids reported in at least two independent studies within each study type. Positive percentages indicate predominant increases, whereas negative percentages indicate predominant decreases.

Amino Acid	Animal Studies		Human Studies		Directional Consistency (%)	
	Blood_↑	Blood_↓	Blood_↑	Blood_↓	Animal Studies	Human Studies
Ala	5	4	1	5	55.56	−83.33
Arg	2	0	3	4	100.00	−57.14
Asp	4	4	2	5	50.00	−71.43
Asn	0	1	0	1	/	/
Cys	0	0	0	5	/	−100.00
GABA	0	1	0	3	/	−100.00
Gln	5	2	2	4	71.43	−66.67
Glu	8	8	7	3	50.00	70.00
Gly	6	3	0	3	66.67	−100.00
His	0	1	0	5	/	−100.00
Ile	2	4	1	0	−66.67	/
Leu	2	8	0	3	−80.00	−100.00
Lys	1	1	0	4	−50.00	−100.00
Met	2	5	0	5	−71.43	−100.00
Phe	7	4	1	7	63.64	−87.50
Pro	0	8	2	1	−100.00	66.67
Ser	3	5	3	2	−62.50	60.00
Thr	1	4	2	1	−80.00	66.67
Trp	3	13	1	5	−81.25	−83.33
Tyr	1	7	0	6	−87.50	−100.00
Val	2	7	2	7	−77.78	−77.78
OH-Trp	0	2	1	1	−100.00	50.00
OH-Pro	1	3	0	1	−75.00	/
OH-Glu	0	1	2	0	/	100.00
Pyro-Glu (Oxo-Pro)	0	5	4	4	−100.00	50.00
N-Ac-Asp	2	0	0	0	100.00	/
N-Ac-Gln	2	0	0	0	100.00	/
N-Ac-Glu	1	0	0	0	/	/
N-Ac-Tyr	0	1	0	0	/	/
N-Ac-Trp	0	1	0	0	/	/

**Table 2:** Vote-counting analysis of significantly increased and decreased amino acids reported in CSF, hippocampus (HIPP), and prefrontal cortex (PFC). Arrows in the column headings indicate the direction of amino acid changes (↑ increased; ↓ decreased), while the numbers represent the number of studies reporting each direction of change. Directional consistency (%) was calculated for amino acids reported in at least two independent studies within each central compartment and study type. Positive percentages indicate predominant increases, whereas negative percentages indicate predominant decreases.

Amino Acid	CSF_↑	CSF_↓	HIPP_↑	HIPP_↓	PFC_↑	PFC_↓	Directional Consistency (%)		
							CSF	HIPP	PFC
Ala	1	0	2	−2	3	−1	/	50.00	75.00
Arg	0	0	0	−1	0	0	/	/	/
Asp	1	0	2	−5	0	−1	/	−71.43	/
Asn	0	0	2	0	1	0	/	100.00	/
Cys	0	0	2	0	0	0	/	100.00	/
GABA	0	0	0	−5	1	−2	/	−100.00	−66.67
Gln	0	0	1	−5	1	−1	/	−83.33	50.00
Glu	0	0	6	−2	0	−1	/	75.00	/
Gly	1	0	2	−2	0	0	/	50.00	/
His	0	0	0	0	0	0	/	/	/
Ile	0	0	0	−2	0	−1	/	−100.00	/
Leu	0	0	1	−1	0	0	/	50.00	/
Lys	0	0	0	0	0	0	/	/	/
Met	0	0	1	−2	0	−1	/	−66.67	/
Phe	0	0	1	−4	0	−1	/	−80.00	/
Pro	1	0	0	−2	0	−1	/	−100.00	/
Ser	0	0	2	−2	0	0	/	50.00	/
Thr	1	0	1	−1	0	0	/	50.00	/
Trp	0	0	1	−3	0	−1	/	−75.00	/
Tyr	0	0	0	−3	0	−1	/	−100.00	/
Val	0	0	1	−2	0	0	/	−66.67	/
OH-Trp	0	0	1	0	0	0	/	/	/
OH-Pro	0	0	0	0	0	−1	/	/	/
Pyro-Glu (Oxo-Pro)	0	0	1	−1	0	0	/	50.00	/
N-Ac-Asp	0	0	3	−4	1	−1	/	−57.14	50.00
N-Ac-Trp	0	0	0	0	0	−3	/	/	−100.00
N-Ac-Leu	0	0	0	0	1	−1	/	/	50.00
N-Ac-His	0	0	0	0	0	−1	/	/	/

## Data Availability

The raw data supporting the conclusions of this article will be made available by the authors on request.

## Supplementary Materials

The following supporting information can be downloaded at: https://www.mdpi.com/article/10.3390/ijms27219847/s1.

## Abbreviations

The following abbreviations are used in this manuscript:

NLP	natural language processing
FDR	false discovery rate
VIP	variable importance in projection
BBB	blood–brain barrier
CSF	cerebrospinal fluid
ML	machine learning
GC	gas chromatography
LC	liquid chromatography
NMR	nuclear magnetic resonance
HIPP	hippocampus
PFC	prefrontal cortex
GABA	γ-aminobutyric acid
SAM	S-adenosylmethionine
CNS	central nervous system
SAH	S-adenosylhomocysteine
THF	tetrahydrofolate
DA	dopamine
NE	norepinephrine
5-HT	serotonin
GSH	glutathione
BCAA	branched-chain amino acid
NAA	N-acetyl aspartate
LAT1	large neutral amino acid transporter 1
CUMS	chronic unpredictable mild stress
CAT	cationic amino acid transporter
MDD	major depressive disorder
CSDS	chronic social defeat stress
B^0^AT2	B^0^ amino acid transporter 2
EAAT	excitatory amino acid transporter
ASCT	alanine-serine-cysteine transporter
