# Supplementary material for: Compartment-Specific Amino Acid Dysregulation in Depression: Integrating Non-Targeted Metabolomics with Brain Barrier Biology
Source: Int J Mol Sci. 2026 Nov 3;27(21):9847. doi: 10.3390/ijms27219847 (PMC13763637; doi:10.3390/ijms27219847)
Supplement: Supplementary file 1 [file ijms-27-09847-s001.zip › Supplementary Figures.pdf]

Supplementary figures:

Title: Compartment-Specific Amino Acid Dysregulation in Depression: Integrating Non-Targeted Metabolomics with Brain Barrier Biology

Authors: Ivana Perić*, Andrijana Stanisavljević Ilić, Neda Đorđević, Nevena Todorović Vukotić

Department of Molecular Biology and Endocrinology, Vinča Institute of Nuclear Sciences—National Institute of the Republic of Serbia, University of Belgrade, Mike Petrovića Alasa 12-14, 11351 Belgrade, Serbia

*Corresponding author:

E-mail: ivanap@vinca.rs

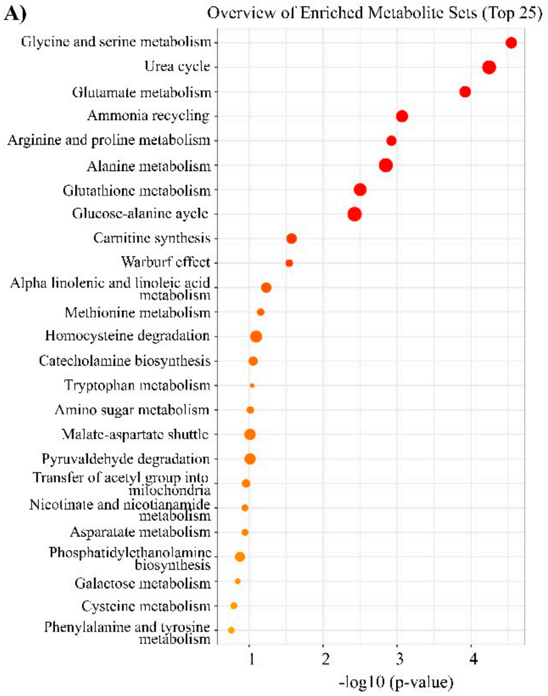

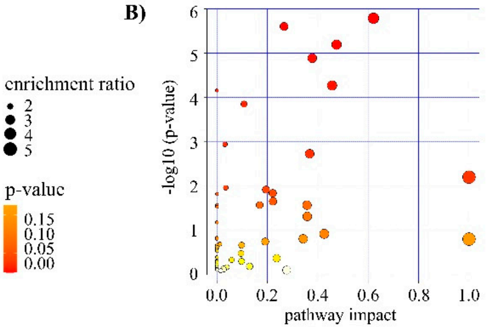

	Total	Hits	p-value	FDR	Impact
Alanine, aspartate and glutamate metabolism	28	8	1.64E-06	0.0001	0.6210
Arginine byiosinthesis	14	6	2.51E-06	0.0001	0.2660
Glycine, serine and threonine metabolism	33	8	6.45E-06	0.0002	0.4744
Arginine and proline metabolism	36	8	1.30E-05	0.0003	0.3779
Glyoxylate and dicarboxylate metabolism	32	7	5.37E-05	0.0009	0.4567
Valine, leucine and isoleucine biosynthesis	8	4	6.96E-05	0.0009	0
One carbon pool by folate	26	6	0.0001	0.0016	0.1071
Butanoate metabolism	15	4	0.0011	0.0114	0.0318
Glutathione metabolism	28	5	0.0019	0.0165	0.3678
Phenylalanine, tyrosine and tryptophan biosynthesis	4	2	0.0063	0.0500	1

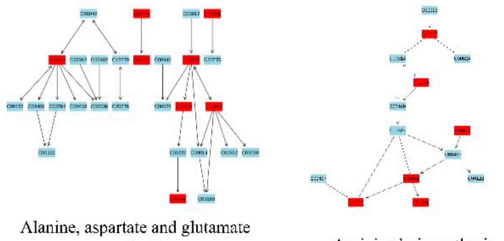

Figure S1. Graphical representation of metabolite set enrichment and pathway analysis results for blood. A) Top 25 enriched metabolite sets identified through over-representation analysis. B) Pathway analysis results, highlighting the two most significantly affected pathways: Alanine, aspartate, and glutamate metabolism (FDR-adjusted p-value = 0.0001; impact = 0.6210) and Arginine biosynthesis (FDR-adjusted p-value = 0.0001 × 10^{-6}; impact = 0.2660).

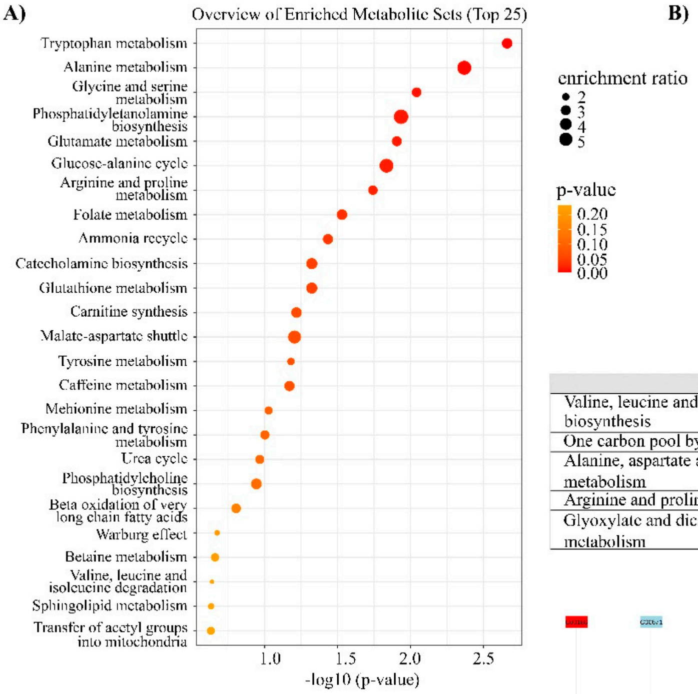

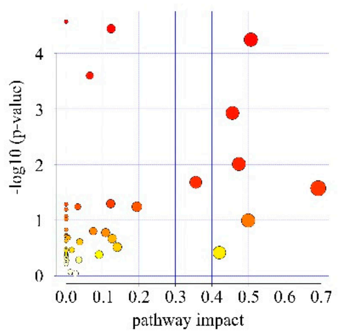

	Total	Hits	p-value	FDR	Impact
Valine, leucine and isoleucine biosynthesis	8	4	2.72E-05	0.0015	0
One carbon pool by folate	26	6	3.64E-05	0.0015	0.1235
Alanine, aspartate and glutamate metabolism	28	6	5.72E-05	0.0015	0.5072
Arginine and proline metabolism	36	6	0.0003	0.0051	0.0651
Glyoxylate and dicarboxylate metabolism	32	5	0.0012	0.0191	0.4567

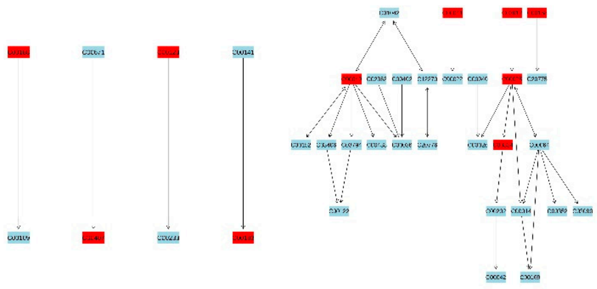

Valine, leucine and isoleucine biosynthesis

Alanine, aspartate and glutamate metabolism

Figure S2. Graphical representation of metabolite set enrichment and pathway analysis results for CSF. A) Top 25 enriched metabolite sets identified through over-representation analysis. B) Pathway analysis results, highlighting two pathways: Valine, leucine and isoleucine biosynthesis (FDR-adjusted p-value = 0.0015; impact = 0) and Alanine, aspartate, and glutamate metabolism (FDR-adjusted p-value = 0.0015; impact = 0.5072).

A)

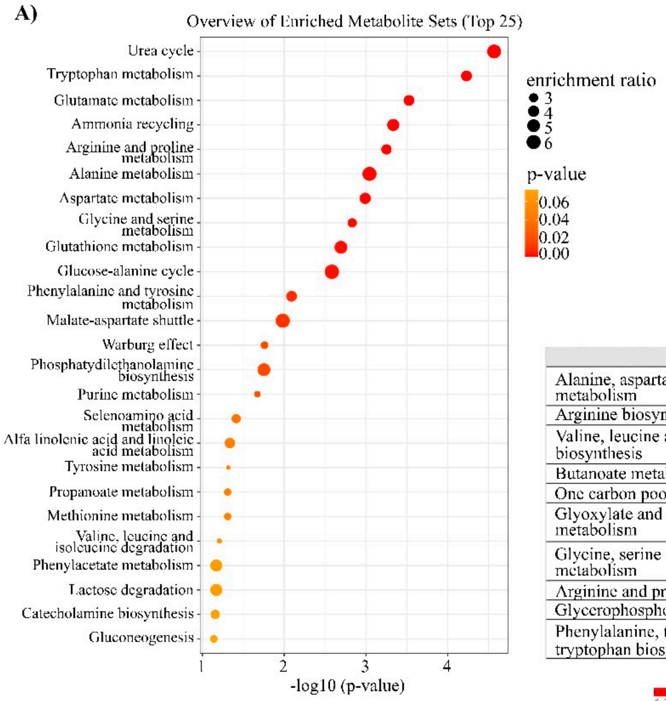

B)

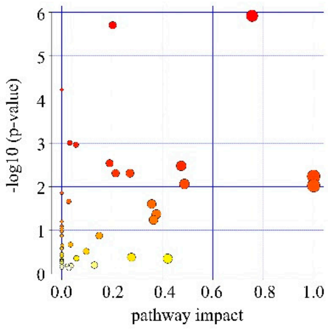

	Total	Hits	p-value	FDR	Impact
Alanine, aspartate and glutamate metabolism	28	8	1.21E-06	7.93E-05	0.7556
Arginine biosynthesis	14	6	1.98E-06	7.93E-05	0.2021
Valine, leucine and isoleucine biosynthesis	8	4	5.96E-05	0.0016	0
Butanoate metabolism	15	4	0.0010	0.0175	0.0318
One carbon pool by folate	26	5	0.0011	0.0175	0.0564
Glyoxylate and dicarboxylate metabolism	32	5	0.0029	0.0380	0.1900
Glycine, serine and threonine metabolism	33	5	0.0033	0.0380	0.4744
Arginine and proline metabolism	36	5	0.0049	0.0437	0.2140
Glycerophospholipid metabolism	36	5	0.0049	0.0437	0.2706
Phenylalanine, tyrosine and tryptophan biosynthesis	4	2	0.0058	0.0464	1

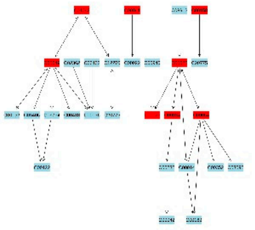

Alanine, aspartate and glutamate metabolism

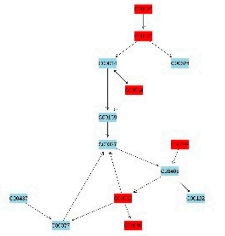

Arginine biosynthesis

Figure S3. Graphical representation of metabolite set enrichment and pathway analysis results for hippocampus. A) Top 25 enriched metabolite sets identified through over-representation analysis. B) Pathway analysis results, highlighting two pathways: Alanine, aspartate, and glutamate metabolism (FDR-adjusted p-value = 7.93×10^{-5}; impact = 0.7556) and Arginine biosynthesis (FDR-adjusted p-value = 7.93×10^{-5}; impact = 0.2021).

A)

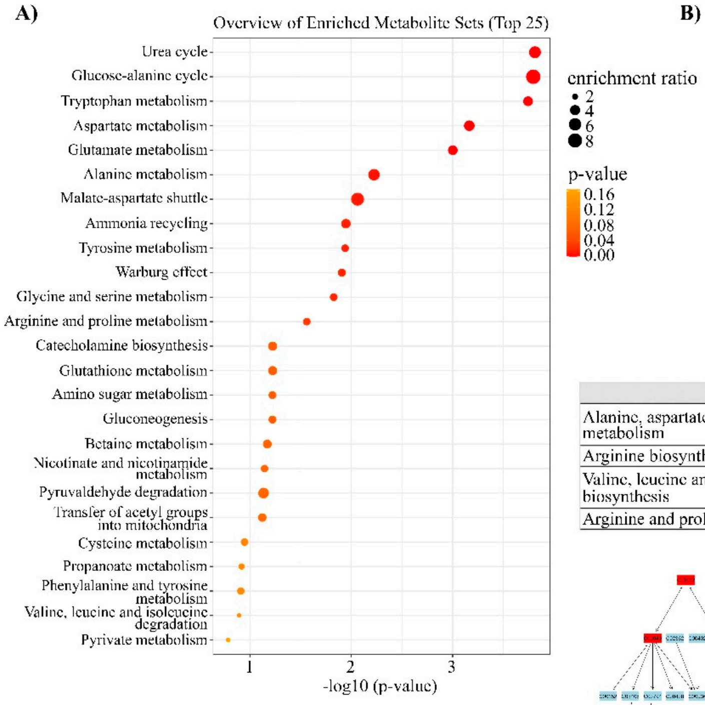

B)

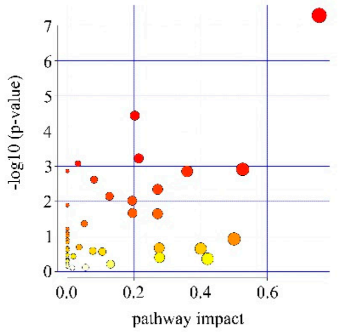

	Total	Hits	p-value	FDR	Impact
Alanine, aspartate and glutamate metabolism	28	6	3.16E-05	0.0025	0.7075
Arginine biosynthesis	14	4	0.0002	0.0093	0.2021
Valine, leucine and isoleucine biosynthesis	8	3	0.0006	0.0173	0
Arginine and proline metabolism	36	5	0.0013	0.0260	0.2140

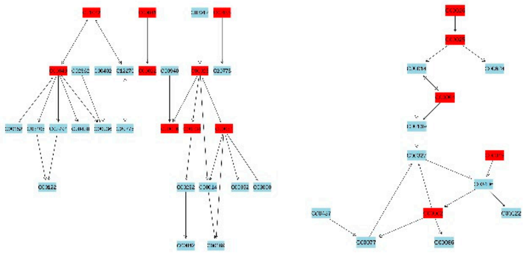

Alanine, aspartate and glutamate metabolism

Arginine biosynthesis

Figure S4. Graphical representation of metabolite set enrichment and pathway analysis results for prefrontal cortex. A) Top 25 enriched metabolite sets identified through over-representation analysis. B) Pathway analysis results, highlighting two pathways: Alanine, aspartate, and glutamate metabolism (FDR-adjusted p-value = 0.0025; impact = 0.7075) and Arginine biosynthesis (FDR-adjusted p-value = 0.0093; impact = 0.2021).
